# Individual Barriers to an Active Lifestyle at Older Ages Among Whitehall II Study Participants After 20 Years of Follow-up

**DOI:** 10.1001/jamanetworkopen.2022.6379

**Published:** 2022-04-07

**Authors:** Mathilde Chen, Manasa S. Yerramalla, Vincent T. van Hees, Mikaela Bloomberg, Benjamin Landré, Aurore Fayosse, Mohamed Amine Benadjaoud, Séverine Sabia

**Affiliations:** 1 Centre of Research in Epidemiology and Statistics, Inserm U1153, Epidemiology of Ageing and Neurodegenerative Diseases, Université de Paris, Paris, France; 2 Accelting, Almere, the Netherlands; 3 Department of Epidemiology and Public Health, University College London, United Kingdom; 4 Department of Radiobiology and Regenerative Medicine, Institute for Radiological Protection and Nuclear Safety, Fontenay-Aux-Roses, France

## Abstract

**Importance:**

Identification of individual-level barriers associated with decreased activity in older age is essential to inform effective strategies for preventing the health outcomes associated with high sedentary behavior and lack of physical activity during aging.

**Objective:**

To assess cross-sectional and prospective associations of a large set of factors with objectively assessed sedentary time and physical activity at older age.

**Design, Setting, and Participants:**

This population-based cohort study was conducted among participants in the Whitehall II accelerometer substudy with accelerometer data assessed in 2012 to 2013. Among 4880 participants invited to the accelerometer substudy, 4006 individuals had valid accelerometer data. Among them, 3808 participants also had factors assessed in 1991 to 1993 (mean [SD] follow-up time, 20.3 [0.5] years), 3782 participants had factors assessed in 2002 to 2004 (mean [SD] follow-up time, 9.1 [0.3] years), and 3896 participants had factors assessed in 2012 to 2013 (mean follow up time, 0 years). Data were analyzed from May 2020 through July 2021.

**Exposures:**

Sociodemographic factors (ie, age, sex, race and ethnicity, occupational position, and marital status), behavioral factors (ie, smoking, alcohol intake, and fruit and vegetable intake), and health-related factors (ie, body mass index, 36-Item Short Form Health Survey (SF-36) physical and mental component summary scores [PCS and MCS], and number of chronic conditions) were assessed among 3808 individuals in 1991 to 1993; 3782 individuals in 2002 to 2004; and 3896 individuals in 2012 to 2013. High alcohol intake was defined as more than 14 units of alcohol per week, and high fruit and vegetable intake was defined as twice daily or more.

**Main Outcomes and Measures:**

Accelerometer-assessed time spent in sedentary behavior, light-intensity physical activity (LIPA), and moderate to vigorous physical activity (MVPA) in 2012 to 2013 were analyzed in 2021 using multivariate linear regressions.

**Results:**

A total of 3896 participants (986 [25.3%] women; age range, 60-83 years; mean [SD] age, 69.4 [5.7] years) had accelerometer data and exposure factors available in 2012 to 2013. Older age, not being married or cohabiting, having overweight, having obesity, more chronic conditions, and poorer SF-36 PCS, assessed in midlife or later life, were associated with increased sedentary time at the expense of time in physical activity. Mean time differences ranged from 9.8 min/d (95% CI, 4.1 to 15.6 min/d) of sedentary behavior per 10-point decrease in SF-36 PCS to 51.4 min/d (95% CI, 37.2 to65.7 min/d) of sedentary behavior for obesity vs reference range weight, from −6.2 min/d (95% CI, −8.4 to −4.1 min/d) of LIPA per 5 years of age to −28.0 min/d (95% CI, −38.6 to −17.4 min/d) of LIPA for obesity vs reference range weight, and from −5.3 min/d (95% CI, −8.2 to −2.4 min/d) of MVPA per new chronic condition to −23.4 min/d (95% CI, −29.2 to −17.6 min/d) of MVPA for obesity vs reference range weight in 20-year prospective analyses for men. There was also evidence of clustering of behavioral factors: high alcohol intake, high fruit and vegetable consumption, and no current smoking were associated with decreased sedentary time (mean time difference in cross-sectional analysis in men: −12.7 min/d [95% CI, −19.8 to −5.5 min/d]; −6.0 min/d [95% CI, −12.3 to −0.2]; and −37.4 min/d [95% CI, − 56.0 to −18.8 min/d], respectively) and more physical activity.

**Conclusions and Relevance:**

This study found a large range of individual-level barriers associated with a less active lifestyle in older age, including sociodemographic, behavioral, and health-related factors. These barriers were already evident in midlife, suggesting the importance of early implementation of targeted interventions to promote physical activity and reduce sedentary time.

## Key Points

**Question:**

What are the long-term individual-level barriers of an active lifestyle in older age?

**Findings:**

In this cohort study of 3896 older adults with accelerometer data, barriers associated with decreased physical activity and increased sedentary behavior in later life, already evident in midlife, were older age, living alone, obesity, morbidities, and poor physical functioning. In older age, there was also evidence of clustering of behavioral factors, given that no current smoking, eating more fruits and vegetables, and drinking more alcohol were associated with decreased sedentary time and more physical activity.

**Meaning:**

These findings suggest that midlife implementation of targeted policies integrating all physical activity components and other healthy behaviors may be an effective approach to promote an active lifestyle in older age.

## Teaser

This cohort study examines sociodemographic, behavioral, and health-related factors associated with sedentary behavior and different intensities of physical activity in older adults after 20 years of follow-up.

## Introduction

Physical activity is key for healthy aging, associated with prevention of chronic diseases^1^ and mortality^2^ and maintenance of several body functions.^3^ Sedentary behavior is increasingly suggested to be associated with detrimental health outcomes independent of physical activity.^2^ However, adherence to the recommended 150 minutes per week of moderate to vigorous physical activity (MVPA)^4^ and less than 8 hours of sedentary behavior per day^5^ is low, particularly among older adults.^6,7^

Various environmental and personal factors are associated with participation in physical activity at older ages.^8^ This has led to community-level interventions to provide supportive environments to reduce sedentary behavior and increase engagement in physical activity,^9^ such as increasing green space and safe walking paths. However, current recommendations mainly apply the one-size-fits-all approach, ignoring individual-level barriers to an active lifestyle. Identification of these factors is important to inform future interventions and prevention policies that could target subgroups likely to be inactive at older ages.^10^ Whether such strategies should focus on older adults or individuals earlier in life remains unclear given the paucity of prospective studies in this domain.^11^

Previous studies on factors associated with physical activity and sedentary behavior mainly relied on self-reported measures. These measures, although informative, are prone to recognition and memory biases, especially in older adults,^12^ and do not capture all forms of physical activity, such as light-intensity physical activity (LIPA).^13^ Accelerometers are increasingly used to objectively assess time accumulated across the full range of activity intensities and levels (ie, sedentary behavior, LIPA, and MVPA).^11^ However, evidence on factors associated with accelerometer-assessed activity levels in older adults is hampered by at least 1 of the following limitations: cross-sectional design,^14,15,16,17^ small sample size,^18,19,20^ or emphasis on a few factors, ignoring the complexity of individual-level barriers to an active lifestyle.^11^ Apart from a few cross-sectional studies describing the entire distribution of activity intensity in older adults by sex, age, or employment status,^21,22,23^ most studies have examined factors associated with total physical activity, time in sedentary behavior, or MVPA,^18,19,24^ ignoring their codependency. Accordingly, we examined cross-sectional and prospective associations of sociodemographic, behavioral, and health-related factors with accelerometer-assessed activity intensity distribution and time in different activity levels in older adults.

## Methods

This cohort study follows the Strengthening the Reporting of Observational Studies in Epidemiology (STROBE) reporting guideline. At each wave of data collection, participants provided informed written consent and research ethics approval was obtained from the University College London ethics committee.

### Study Participants

The Whitehall II prospective cohort study was established in 1985 to 1988 among 10 308 British civil servants (6895 [66.9%] men) aged 35 to 55 years.^25^ Since study baseline, sociodemographic, behavioral, and health-related factors were assessed using questionnaires and clinical examinations approximately every 4 to 5 years. An accelerometer measure was added to the 2012 to 2013 wave of data collection for participants seen at the London clinic and those living in the southeastern regions of England who underwent clinical examination at home.

### Outcome: Accelerometer-Assessed Physical Activity and Sedentary Behavior

At the 2012 to 2013 clinical examination, participants were asked to wear a triaxial accelerometer (GENEActiv Original; Activinsights) on their nondominant wrist for 9 days. Accelerometer data, sampled at 85.7 Hz, were processed using the GGIR package version 2.0-0 for R statistical software version 3.6.1 (R Project for Statistical Computing),^26^ expressed relative to gravity (1 *g* = 9.8 m/s^2^), and corrected for calibration error.^26^ Waking periods (ie, periods between waking and sleep onset) for each day were identified using a validated algorithm for sleep detection.^26^ Data from waking periods from day 2 through day 8 were retained, resulting in 7 days of data. Participants with valid data (ie, wear time ≥two-thirds of waking hours for at least 2 weekdays and 2 weekend days^27^) were included in analyses. Valid data were corrected for nonwear time based on a previously reported algorithm.^26^ For each participant, daily activity intensity distribution during waking periods was derived based on time in 0.005 *g* intervals over the full acceleration range (eMethods 1 in the Supplement). Daily time in sedentary behavior, LIPA, and MVPA during waking periods was computed based on a mean acceleration of less than 0.04 *g*, 0.04*g* to 0.099 *g*, and 0.1 *g* or more, respectively,^28^ over 60-second epochs.

### Exposures: Sociodemographic, Behavioral, and Health-Related Factors

Exposure variables were considered at 3 time points (ie, 2012-2013, 2002-2004, and 1991-1993) to assess whether factors associated with physical activity and sedentary behavior in older age were similar when assessed concurrently or 9 or 20 years before the measure of activity levels. Data were drawn from questionnaires, clinical evaluations, and electronic health records. Sociodemographic factors included age, sex, self-reported race and ethnicity, marital status, and last occupational position, which was defined as low (clerical and support positions), intermediate (professional and executive positions), and high (administrative position), coded 1, 0.5, and 0, respectively, and entered as an ordinal variable; this is a comprehensive marker of socioeconomic position in the British Civil Service that reflects salary, social status, and level of responsibility at work. Available options for self-reported race and ethnicity were Black, South Asian, White, and other. Race and ethnicity were categorized as White individuals and members of racial and ethnic minority groups (ie, Black individuals, South Asian individuals, and individuals with other race or ethnicity) owing to small numbers in these groups. The Whitehall II study was designed to study social disparities in health, and race and ethnicity were included among these sociodemographic factors. Behavioral factors were smoking status (ie, never, past, and current), alcohol intake (ie none [0 units/wk], moderate [1-14 units/wk], and high [>14 units/wk]), and fruit and vegetable intake (ie, <2 times/d or ≥2 times/d). Health-related factors included body mass index (BMI; calculated as weight in kilograms divided by height in meters squared; categorized as reference range [<25], overweight [25-29.9], and obese [≥30]), 36-Item Short Form Health Survey (SF-36)^29^ physical and mental component summary (PCS and MCS) scores, and number of chronic conditions, including hypertension, diabetes, coronary heart disease, stroke, heart failure, arthritis, cancer, depression, dementia, Parkinson disease, and chronic obstructive pulmonary disease. Further details on these variables are provided in eMethods 2 in the Supplement.

### Statistical Analysis

We conducted 3 sets of analyses. First, sex differences in activity intensity distribution and time in sedentary behavior, LIPA, and MVPA were assessed using univariate function-on-scalar regression and *t* test, respectively. Second, cross-sectional associations of exposure variables with activity intensity distribution and time in sedentary behavior, LIPA, and MVPA in 2012 to 2013 were examined using multivariate function-on-scalar regression (eMethods 1 in the Supplement) and 3 separate linear regression models, respectively. Models were adjusted for exposure variables and waking time. Owing to significant interaction associations between most factors investigated and sex (eTable 1 in the Supplement), analyses were conducted separately in men and women. Third, the last set of analyses was repeated in prospective analyses using exposure variables assessed in 1991 to 1993 (ie, 20-year follow-up) and 2002 to 2004 (ie, 9-year follow-up).

We conducted 2 sets of sensitivity analyses. First, prospective analyses were additionally adjusted for self-reported MVPA measured concurrently to exposure variables given that physical activity may be a confounding factor in the association between exposure variables and physical activity in later life. Second, multiple imputation (20 imputations) was used to account for missing data in linear regression models.

Significance of associations was examined using *P* values from function-on-scalar regression that accounts for the entire activity intensity distribution. Using Bonferroni correction for multiple testing, 2-sided *P* values < .004 were considered significant (ie, .05 divided by 14 tests per model). Analyses were undertaken using R statistical software version 3.6.1 (R Project for Statistical Computing) and Stata statistical software version 15 (StataCorp). Data were analyzed from May 2020 through July 2021.

## Results

Of 4880 participants invited to participate in the accelerometer substudy, 4006 individuals had valid accelerometer data. Of these participants, 3896 individuals had data on sociodemographic, behavioral, or health-related factors in 2012 to 2013 and were included in cross-sectional analyses (mean [SD] age, 69.4 [5.7] years; age range, 60-83 years; 986 [25.3%] women). There were 86 Black individuals (2.21%), 158 South Asian individuals (4.1%), 3622 White individuals (93.0%), and 30 individuals with other race or ethnicity (0.8%). Prospective analyses included 3808 and 3782 participants with factors assessed in 1991 to 1993 (mean [SD] age, 49.1 [5.7] years; mean [SD] follow-up, 20.3 [0.5] years) and 2002 to 2004 (mean [SD] age, 60.4 [5.7] years; mean [SD] follow-up, 9.1 [0.3] years), respectively. See flowchart in eFigure 1 in the Supplement and characteristics of missing participants in eTable 2 in the Supplement. Characteristics of study participants at each time point are provided in Table 1. Men had a greater mean (SD) time spent in sedentary behavior (721.5 [98.0] min/d vs 708.2 [105.0] min/d; *P* < .001) and MVPA (58.4 [38.4] min/d vs 49.8 [38.2] min/d; *P* < .001) but less in LIPA (205.3 [66.6] min/d vs 224.2 [74.3] min/d; *P* < .001) than women (Table 1). These results are corroborated with the daily activity intensity distribution in men and women, presented in the Figure, A, and their difference in the Figure, B. Time in the 0 to 0.02 *g* acceleration range was increased for men; sex differences decreased progressively when acceleration increased from 0.005 *g* to 0.02 *g* and reversed for acceleration between 0.025 *g* and 0.1 *g*, indicating that women spent more time in this acceleration range. Men spent more time at acceleration greater than 0.1 *g*.

**Table 1.** Characteristics of the Study Population

Characteristic	Participants in 1991-1993, No. (%)		*P* value	Participants in 2002-2004, No. (%)		*P* value	Participants in 2012-2013, No. (%)		*P* value
	Men (n = 2823)	Women (n = 985)		Men (n = 2828)	Women (n = 954)		Men (n = 2910)	Women (n = 986)	
Sociodemographic factor									
Age, mean (SD)	49.0 (5.7)	49.3 (5.8)	.23	60.4 (5.7)	60.4 (5.8)	.89	69.4 (5.7)	69.5 (5.8)	.70
Member of racial or ethnic minority group^a^	153 (5.4)	123 (12.5)	<.001	154 (5.4)	105 (11.0)	<.001	158 (5.4)	116 (11.8)	<.001
Race and ethnicity									
Black	40 (1.4)	55 (5.6)	NA	37 (1.3)	41 (4.3)	NA	40 (1.4)	46 (4.7)	NA
South Asian	99 (3.5)	53 (5.4)	NA	103 (3.6)	49 (5.1)	NA	104 (3.6)	54 (5.5)	NA
White	2670 (94.6)	862 (87.5)	NA	2674 (94.6)	849 (89.0)	NA	2752 (94.6)	870 (88.2)	NA
Other	14 (0.5)	15 (1.5)	NA	14 (0.5)	15 (1.6)	NA	14 (0.5)	16 (1.6)	NA
Occupational position^b^									
High	1426 (50.5)	206 (20.9)	<.001	1592 (56.3)	255 (26.7)	<.001	1657 (56.9)	270 (27.4)	<.001
Intermediate	1286 (45.6)	478 (48.5)		1141 (40.3)	470 (49.3)		1151 (39.6)	485 (49.2)	
Low	111 (3.9)	301 (30.6)		95 (3.4)	229 (24.0)		102 (3.5)	231 (23.4)	
Not married or cohabitating	458 (16.2)	372 (37.8)	<.001	459 (16.2)	420 (44.0)	<.001	511 (17.6)	469 (47.6)	<.001
Behavioral factors									
Smoking status									
Never	1449 (51.3)	577 (58.6)	<.001	1362 (48.2)	541 (56.7)	<.001	1326 (45.6)	549 (55.7)	<.001
Past	1108 (39.2)	298 (30.3)		1286 (45.5)	338 (35.4)		1499 (51.5)	398 (40.4)	
Current	266 (9.4)	110 (11.2)		180 (6.4)	75 (7.9)		85 (2.9)	39 (4.0)	
Alcohol intake, units/wk									
None (0)	358 (12.7)	254 (25.8)	<.001	305 (10.8)	243 (25.5)	<.001	450 (15.5)	327 (33.2)	<.001
Moderate (1-14)	1610 (57.0)	628 (63.8)		1469 (51.9)	574 (60.2)		1643 (56.5)	564 (57.2)	
High (>14)	855 (30.3)	103 (10.5)		1054 (37.3)	137 (14.4)		817 (28.1)	95 (9.6)	
Fruit and vegetable intake <2 daily	564 (20.0)	297 (30.2)	<.001	1091 (38.6)	474 (49.7)	<.001	1613 (55.4)	655 (66.4)	<.001
Health-related factors									
BMI									
Reference range (<25)	1576 (55.8)	548 (55.6)	<.001	1045 (37.0)	404 (42.3)	<.001	1110 (38.1)	401 (40.7)	<.001
Overweight (25-29.9)	1090 (38.6)	314 (31.9)		1391 (49.2)	321 (33.6)		1347 (46.3)	330 (33.5)	
Obese (≥30)	157 (5.6)	123 (12.5)		392 (13.9)	229 (24.0)		453 (15.6)	255 (25.9)	
SF-36, mean (SD)									
MCS score	51.5 (8.1)	49.7 (9.4)	<.001	52.6 (8.4)	51.0 (10.1)	<.001	54.2 (7.6)	52.3 (9.9)	<.001
PCS score	53.5 (5.7)	51.0 (8.1)	<.001	50.6 (7.4)	47.4 (10.0)	<.001	49.0 (8.1)	46.0 (10.6)	<.001
No. of chronic conditions, mean (SD)^c^	0.2 (0.5)	0.2 (0.4)	.03	0.6 (0.8)	0.7 (0.9)	<.001	1.2 (1.0)	1.2 (1.1)	.05
Accelerometer variables, mean (SD), min/d									
Sedentary behavior	NA	NA	NA	NA	NA	NA	721.5 (98.0)	708.2 (105.0)	<.001
LIPA	NA	NA	NA	NA	NA	NA	205.3 (66.6)	224.2 (74.3)	<.001
MVPA	NA	NA	NA	NA	NA	NA	58.4 (38.4)	49.8 (38.2)	<.001

Abbreviations: BMI, body mass index (calculated as weight in kilograms divided by height in meters squared); LIPA, light-intensity physical activity; MCS, mental component summary; MVPA, moderate to vigorous physical activity; NA, not applicable; PCS, physical component summary; SF-36, 36-Item Short Form Health Survey.

^a^ Race and ethnicity were assessed by questionnaire using the response categories Black, South Asian, White, and other. Racial and ethnic minority groups have been combined in analysis owing to small numbers.

^b^ Last occupational position was defined as low (clerical or support positions), intermediate (professional or executive positions), or high (administrative positions), coded as 1, 0.5, and 0, respectively, and entered as an ordinal variable in analyses.

^c^ Chronic conditions include hypertension, diabetes, coronary heart disease, stroke, heart failure, arthritis, cancer, depression, dementia, Parkinson disease, and chronic obstructive pulmonary disease.

**Figure.**
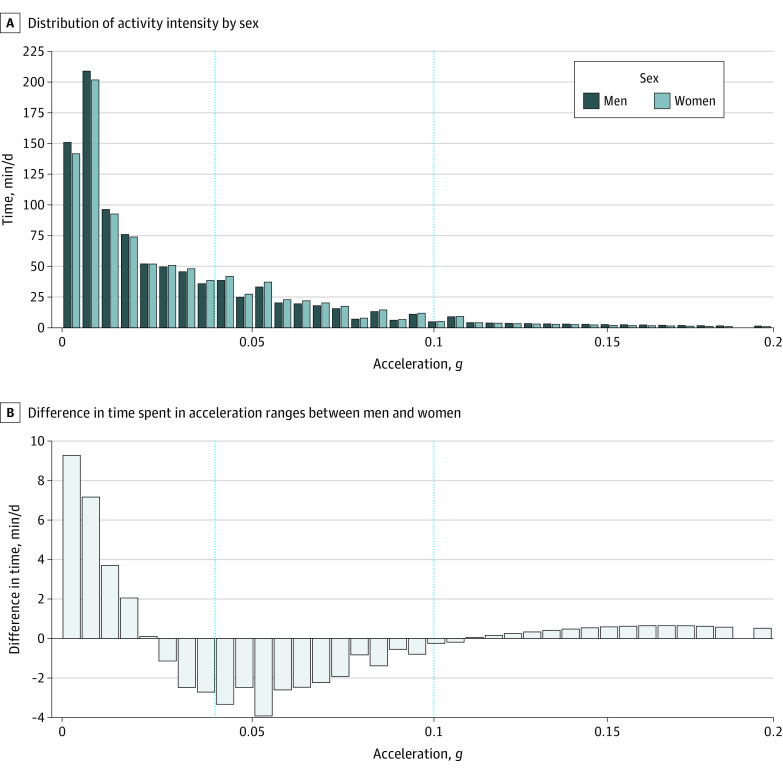
Sex Differences in Activity Intensity Distribution Vertical dotted lines indicate cutoffs between sedentary behavior, light-intensity physical activity (0.04 *g*), and moderate to vigorous physical activity (0.1 *g*).

In cross-sectional analyses among 2910 men of sociodemographic factors examined, older age (mean time difference per 5 years of age, 15.9 min/d [95% CI, 13.1 to 18.8 min/d]; *P* < .001), membership in a racial or ethnic minority group (mean time difference vs White individuals, 24.9 min/d [95% CI, 10.9 to 38.9 min/d]; *P* < .001), and absence of a partner (mean time difference, 11.4 min/d [95% CI, 3.2 to 19.7 min/day]; *P* < .001) were associated with increased time in sedentary behavior at the expense of time in LIPA or MVPA (Table 2; eFigure 2 in the Supplement). By contrast, low occupational position was associated with less time in sedentary behavior vs high occupational position (mean time difference, −15.7 min/d [95% CI, −27.2 to −4.2 min/d]) and more time in physical activity (mean time difference for LIPA: 14.1 min/d [95% CI, 5.4 to 22.8 min/d] and MVPA: 1.6 min/d [95% CI, −3.0 to 6.3 min/d]) (*P* < .001). Differences in sedentary time were similar per 5-year increase in age between higher and lower occupational positions and between those without and with partners, although the redistribution of time to other activity levels differed for each factor (Table 2). For example, the 11-minute increase in sedentary behavior for individuals without a partner was mainly at the expense of LIPA (mean time difference, −13.8 min/d [95% CI, −20.0 to −7.6 min/d]; *P* < .001), whereas increase in sedentary time per 5-year of age was mainly at the expense of time in MVPA (mean time difference, −10.4 min/d [95% CI, −11.5 to −9.2 min/d]; *P* < .001), representing nearly 50% of the daily recommended 21 minutes of MVPA. All behavioral factors were associated with the activity intensity distribution (Table 2; eFigure 3 in the Supplement). The largest difference was found for current smokers who spent a mean 37.4 more minutes per day sedentary (95% CI, 18.8 to 56.0 min/d) than never smokers, at the expense of a mean 23.3 min/d (95% CI, −37.3 to −9.3 min/d) and 14.1 min/d (95% CI,−21.6 to −6.6 min/d) fewer minutes in LIPA and MVPA, respectively (*P* < .001) (Table 2). The latter difference was two-thirds of the recommended MVPA duration. Higher alcohol intake was associated with less sedentary time (mean time difference, −12.7 min/d [95% CI, −19.8 to −5.5 min/d]) and more LIPA (mean time difference, 9.3 min/d [95% CI, 3.9 to 14.7 min/d]) and MVPA (mean time difference, 3.4 min/d [95% CI, 0.5 to 6.3 min/ d]) (*P* < .001). Among health-related factors, obesity, poorer physical functioning, and an increased number of chronic conditions were associated with the activity intensity distribution (Table 2; eFigure 4 in the Supplement). The largest mean time difference was found for obese vs reference range BMI groups: 50.7 min/d (95% CI, 41.2 to 60.3 min/d) for sedentary behavior, −28.6 min/d (95% CI, −35.8 to −21.4 min/d) for LIPA, and −22.1 min/d (95% CI, −25.9 to −18.2 min/d) for MVPA (*P* < .001), the latter corresponding to the recommended MVPA duration (Table 2).

**Table 2.** Cross-sectional Association of Factors With Sedentary Behavior and Physical Activity

Factor	Men (n = 2910)				Women (n = 986)				*P* value for interaction by sex^c^
	Mean time difference (95% CI), min/d^a^			*P* value for activity distribution^b^	Mean time difference (95% CI), min/d^a^			*P* value for activity distribution^b^	
	SB	LIPA	MVPA		SB	LIPA	MVPA		
Sociodemographic factor									
Age, per 5 y	15.9 (13.1 to 18.8)	−5.6 (−7.7 to −3.4)	−10.4 (−11.5 to −9.2)	<.001^d^	16.2 (10.9 to 21.6)	−6.3 (−10.6 to −2.1)	−9.9 (−11.9 to −7.9)	<.001^d^	.69
Member of racial or ethnic minority group^e^	24.9 (10.9 to 38.9)	−18.7 (−29.3 to −8.1)	−6.2 (−11.8 to −0.5)	<.001^d^	11.3 (−7.6 to 30.2)	−6.1 (−21.0 to 8.8)	−5.1 (−12.2 to 1.9)	<.001^d^	<.001^d^
Lower occupational position^f^	−15.7 (−27.2 to −4.2)	14.1 (5.4 to 22.8)	1.6 (−3.0 to 6.3)	<.001^d^	−15.0 (−32.5 to 2.5)	13.5 (−0.2 to 27.3)	1.4 (−5.0 to 7.9)	.05	<.001^d^
Not married or cohabitating	11.4 (3.2 to 19.7)	−13.8 (−20.0 to −7.6)	2.3 (−1.0 to 5.7)	<.001^d^	11.5 (−0.3 to 23.2)	−12.0 (−21.3 to −2.8)	0.6 (−3.8 to 4.9)	<.001^d^	.03
Behavioral factor									
Smoking status									
Never	1 [Reference]	1 [Reference]	1 [Reference]	NA	1 [Reference]	1 [Reference]	1 [Reference]	NA	NA
Past	−7.3 (−13.6 to −0.9)	4.7 (−0.1 to 9.5)	2.6 (0.1 to 5.2)	<.001^d^	−0.4 (−12.3 to 11.5)	−0.2 (−9.6 to 9.2)	0.6 (−3.8 to 5.1)	.22	<.001^d^
Current	37.4 (18.8 to 56.0)	−23.3 (−37.3 to −9.3)	−14.1 (−21.6 to −6.6)	<.001^d^	26.4 (−3.3 to 56.0)	−11.5 (−34.9 to 11.9)	−14.9 (−25.9 to −3.9)	.02	<.001^d^
Alcohol intake^g^									
None	9.4 (0.5 to 18.4)	−4.9 (−11.7 to 1.8)	−4.5 (−8.1 to −0.9)	<.001^d^	4.2 (−9.0 to 17.5)	−2.8 (−13.2 to 7.7)	−1.4 (−6.4 to 3.5)	<.001^d^	.27
Moderate	1 [Reference]	1 [Reference]	1 [Reference]	NA	1 [Reference]	1 [Reference]	1 [Reference]	NA	NA
High	−12.7 (−19.8 to −5.5)	9.3 (3.9 to 14.7)	3.4 (0.5 to 6.3)	<.001^d^	−10.6 (−30.2 to 9.1)	3.0 (−12.5 to 18.5)	7.6 (0.3 to 14.9)	<.001^d^	.97
Fruit and vegetables intake <2 daily	6.0 (−0.2 to 12.3)	−4.2 (−8.9 to 0.5)	−1.9 (−4.4 to 0.7)	<.001^d^	3.5 (−9.0 to 16.0)	−3.3 (−13.2 to 6.6)	−0.2 (−4.8 to 4.5)	<.001^d^	.58
Health-related factors									
BMI^h^									
Reference range	1 [Reference]	1 [Reference]	1 [Reference]	NA	1 [Reference]	1 [Reference]	1 [Reference]	NA	NA
Overweight	17.4 (10.6 to 24.1)	−7.6 (−12.8 to −2.5)	−9.7 (−12.5 to −7.0)	<.001^d^	17.5 (4.3 to 30.7)	−10.5 (−20.9 to −0.0)	−7.0 (−11.9 to −2.1)	<.001^d^	.85
Obese	50.7 (41.2 to 60.3)	−28.6 (−35.8 to −21.4)	−22.1 (−25.9 to −18.2)	<.001^d^	50.7 (35.7 to 65.7)	−33.8 (−45.6 to −22.0)	−16.9 (−22.4 to −11.3)	<.001^d^	<.001^d^
MCS, per 10-point decrease^i^	4.3 (0.2 to 8.5)	−1.4 (−4.5 to 1.7)	−3.0 (−4.6 to −1.3)	.19	6.7 (1.0 to 12.4)	−4.2 (−8.7 to 0.3)	−2.5 (−4.6 to −0.4)	<.001^d^	<.001^d^
PCS, per 10-point decrease^i^	10.9 (6.8 to 15.0)	−4.2 (−7.3 to −1.1)	−6.8 (−8.4 to −5.1)	<.001^d^	10.2 (4.0 to 16.4)	−5.2 (−10.1 to −0.3)	−5.0 (−7.3 to −2.7)	<.001^d^	.33
No. of chronic conditions, per new condition^j^	6.8 (3.5 to 10.0)	−4.0 (−6.5 to −1.6)	−2.7 (−4.1 to −1.4)	<.001^d^	9.1 (3.4 to 14.7)	−5.2 (−9.6 to −0.7)	−3.9 (−6.0 to −1.8)	<.001^d^	.66

Abbreviations: BMI, body mass index (calculated as weight in kilograms divided by height in meters squared); LIPA, light-intensity physical activity; MCS, mental component summary score from the 36-Item Short Form Health Survey; MVPA, moderate to vigorous physical activity; NA, not applicable; PCS, physical component summary score from the 36-Item Short Form Health Survey; SB, sedentary behavior.

^a^ Estimated from linear regression adjusted for covariates and waking time.

^b^ *P* for association between each factor and activity intensity distribution from function-on-scalar regression adjusted for covariates and waking time.

^c^ *P* from function-on-scalar regression assessing interactions of sex with covariates.

^d^ *P* values < .004 were considered significant according to the Bonferroni correction for multiple testing.

^e^ Race and ethnicity were assessed by questionnaire using the response categories Black, South Asian, White, and other. Racial and ethnic minority groups have been combined in analysis owing to small numbers.

^f^ Last occupational position was defined as low (clerical or support positions), intermediate (professional or executive positions), or high (administrative positions), coded as 1, 0.5, and 0, respectively, and entered as an ordinal variable in analyses.

^g^ Alcohol intake was defined as 0 units/wk for none, 1 to 14 units/wk for moderate, and more than 14 units/wk for high.

^h^ BMI categories were defined as less than 25 for reference range, 25 to 29.9 for overweight, and 30 or more for obese.

^i^ Lower MCS and PCS correspond to poorer mental and physical summary scores, respectively.

^j^ Chronic conditions include hypertension, diabetes, coronary heart disease, stroke, heart failure, arthritis, cancer, depression, dementia, Parkinson disease, and chronic obstructive pulmonary disease.

Among 986 women, all factors were associated with activity intensity distribution, except for past smoking and occupational position (Table 2; eFigures 2, 3, and 4 in the Supplement). Associations were similar for most factors but globally attenuated for women compared with men (Table 2). For example, mean time difference in sedentary activity at the expense of time in LIPA or MVPA among women was 16.2 min/d (95% CI, 10.9-21.6 min/d; *P* < .001) per 5-year increase in age (*P* for interaction by sex = .69).

For men (Table 3) and women (Table 4), prospective associations of exposure variables assessed in 1991 to 1993 and in 2002 to 2004 with activity intensity distribution and time in the different activity levels in 2012 to 2013 were similar to cross-sectional associations, with some exceptions. For example, mean time differences ranged from 9.8 min/d (95% CI, 4.1 to 15.6 min/d; *P* < .001) of sedentary behavior per 10-point decrease in SF-36 PCS to 51.4 min/d (95% CI, 37.2 to 65.7 min/d; *P* < .001) of sedentary behavior for obesity vs reference range weight, from −6.2 min/d (95% CI, −8.4 to −4.1 min/d; *P* < .001) of LIPA per 5 years of age to −28.0 min/d (95% CI, −38.6 to −17.4 min/d; *P* < .001) of LIPA for obesity vs reference range weight, and from −5.3 min/d (95% CI, −8.2 to −2.4 min/d; *P* < .001) of MVPA per new chronic condition to −23.4 min/d (95% CI, −29.2 to −17.6 min/d; *P* < .001) of MVPA for obesity vs reference range weight in 20-year prospective analyses for men (Table 3). Among women, mean time differences ranged from 8.4 min/d (95% CI, 2.0 to 14.8 min/d; *P* = .01) of sedentary behavior per 10-point decrease in SF-36 MCS to 44.8 min/d (95% CI, 26.1 to 63.6 min/d; *P* < .001) of sedentary behavior for obesity vs reference range weight, from −27.8 min/d (95% CI, −42.5 to −13.2 min/d; *P* < .001) of LIPA for obesity vs reference range weight to −6.0 min/d (95% CI, −10.1 to −1.8 min/d; *P* = .0046) of LIPA per 5-year increase in age, and from −17.0 min/d (95% CI, -23.9 to −10.1 min/d; *P* < .001) of MVPA for obesity vs reference range weight to −3.5 min/d (95% CI, −5.8 to −1.1 min/d; *P* = .0037) of MVPA per 10-point decrease in SF-36 MCS in 20-year prospective analyses (Table 4). In the associations for alcohol consumption and current smoking, mean time differences were larger when assessed cross-sectionally. By contrast, in the association between number of chronic conditions and activity levels, mean time differences were larger among men assessed in 1991 to 1993.

**Table 3.** Prospective Association of Factors With Sedentary Behavior and Physical Activity Among Men at 20 Years and 9 Years^a^

Factor	20-y Follow-up (n = 2823)				9-y Follow-up (n = 2828)			
	Mean time difference (95% CI), min/d^b^			*P* value for activity distribution^c^	Mean time difference (95% CI), min/d^b^			*P* value for activity distribution^c^
	SB	LIPA	MVPA		SB	LIPA	MVPA	
Sociodemographic factor								
Age, per 5 y	17.2 (14.4 to 20.1)	−6.2 (−8.4 to −4.1)	−11.0 (−12.2 to −9.8)	<.001^d^	17.5 (14.5 to 20.5)	−6.3 (−8.5 to −4.1)	−11.2 (−12.4 to −10.0)	<.001^d^
Member of racial or ethnic minority group^e^	29.4 (14.8 to 44.0)	−21.3 (−32.2 to −10.4)	−8.1 (−14.0 to −2.1)	<.001^d^	27.7 (13.3 to 42.1)	−20.4 (−31.2 to −9.6)	−7.3 (−13.1 to −1.4)	<.001^c^
Lower occupational position^f^	−11.9 (−23.9 to 0.0)	11.7 (2.7 to 20.6)	0.3 (−4.6 to 5.1)	<.001^d^	−20.2 (−32.2 to −8.2)	16.9 (7.9 to 25.9)	3.3 (−1.6 to 8.2)	<.001^d^
Not married or cohabitating	12.9 (4.0 to 21.8)	−14.1 (−20.8 to −7.5)	1.2 (−2.4 to 4.9)	<.001^d^	11.8 (3.1 to 20.5)	−14.2 (−20.7 to −7.7)	2.4 (−1.1 to 6.0)	<.001^d^
Behavioral factor								
Smoking status								
Never	1 [Reference]	1 [Reference]	1 [Reference]	NA	1 [Reference]	1 [Reference]	1 [Reference]	NA
Past	−8.4 (−15.3 to −1.6)	6.0 (0.9 to 11.1)	2.4 (−0.4 to 5.2)	<.001^d^	−9.2 (−15.8 to −2.6)	6.3 (1.3 to 11.2)	2.9 (0.2 to 5.6)	<.001^d^
Current	18.7 (7.2 to 30.2)	−11.0 (−19.6 to −2.4)	−7.8 (−12.4 to −3.1)	<.001^d^	25.0 (11.6 to 38.3)	−16.3 (−26.3 to −6.2)	−8.7 (−14.1 to −3.3)	<.001^d^
Alcohol intake^g^								
None	5.6 (−4.4 to 15.5)	−2.4 (−9.8 to 5.1)	−3.2 (−7.2 to 0.9)	<.001^d^	9.6 (−1.1 to 20.3)	−6.5 (−14.5 to 1.5)	−3.1 (−7.4 to 1.3)	<.001^d^
Moderate	1 [Reference]	1 [Reference]	1 [Reference]	NA	1 [Reference]	1 [Reference]	1 [Reference]	NA
High	1.3 (−6.0 to 8.6)	−1.1 (−6.5 to 4.3)	−0.2 (−3.2 to 2.8)	<.001^d^	−4.1 (−11.0 to 2.7)	3.9 (−1.3 to 9.0)	0.3 (−2.5 to 3.1)	<.001^d^
Fruit and vegetable intake <2 daily	13.2 (5.2 to 21.2)	−7.8 (−13.8 to −1.9)	−5.3 (−8.6 to −2.1)	<.001^d^	8.0 (1.4 to 14.5)	−4.0 (−9.0 to 0.9)	−3.9 (−6.6 to −1.3)	<.001^d^
Health-related factors								
BMI^h^								
Reference range	1 [Reference]	1 [Reference]	1 [Reference]	NA	1 [Reference]	1 [Reference]	1 [Reference]	NA
Overweight	17.5 (10.8 to 24.3)	−8.8 (−13.8 to −3.8)	−8.7 (−11.5 to −6.0)	<.001^d^	12.6 (5.7 to 19.4)	−5.2 (−10.4 to −0.1)	−7.3 (−10.1 to −4.6)	<.001^d^
Obese	51.4 (37.2 to 65.7)	−28.0 (−38.6 to −17.4)	−23.4 (−29.2 to −17.6)	<.001^d^	45.4 (35.2 to 55.5)	−25.0 (−32.7 to −17.4)	−20.3 (−24.4 to −16.2)	<.001^d^
MCS, per 10-point decrease^i^	2.2 (−1.9 to 6.2)	−0.5 (−3.5 to 2.6)	−1.7 (−3.3 to 0.0)	<.001^d^	2.5 (−1.4 to 6.3)	−0.7 (−3.6 to 2.2)	−1.8 (−3.4 to −0.2)	.23
PCS, per 10-point decrease^i^	9.8 (4.1 to 15.6)	−4.2 (−8.5 to 0.1)	−5.6 (−8.0 to −3.3)	<.001^d^	7.9 (3.4 to 12.3)	−3.5 (−6.8 to −0.2)	−4.4 (−6.2 to −2.6)	<.001^d^
No. of chronic conditions, per new condition^j^	13.2 (6.1 to 20.4)	−7.9 (−13.3 to −2.6)	−5.3 (−8.2 to −2.4)	<.001^d^	13.4 (9.0 to 17.7)	−7.9 (−11.2 to −4.6)	−5.4 (−7.2 to −3.7)	<.001^d^

Abbreviations: BMI, body mass index (calculated as weight in kilograms divided by height in meters squared); LIPA, light-intensity physical activity; MCS, mental component summary score from the 36-Item Short Form Health Survey; MVPA, moderate to vigorous physical activity; NA, not applicable; PCS, physical component summary score from the 36-Item Short Form Health Survey; SB, sedentary behavior.

^a^ Associations are between factors as measured in 1991 to 1993 (for 20 years of follow-up) or 2002 to 2004 (for 9 years of follow-up) and outcomes measured in 2012 to 2013.

^b^ Estimated from linear regression adjusted for covariates and waking time.

^c^ *P* for association between each factor and activity intensity distribution from function-on-scalar regression adjusted for covariates and waking time.

^d^ *P* values < .004 were considered significant according to the Bonferroni correction for multiple testing.

^e^ Race and ethnicity were assessed by questionnaire using the response categories Black, South Asian, White, and other. Racial and ethnic minority groups have been combined in analysis owing to small numbers.

^f^ Last occupational position was defined as low (clerical or support positions), intermediate (professional or executive positions), or high (administrative positions), coded as 1, 0.5, and 0, respectively, and entered as an ordinal variable in analyses.

^g^ Alcohol intake was defined as 0 units/wk for none, 1 to 14 units/wk for moderate, and more than 14 units/wk for high.

^h^ BMI categories were defined as less than 25 for reference range, 25 to 29.9 for overweight, and 30 or more for obese.

^i^ Lower MCS and PCS correspond to poorer mental and physical summary score, respectively.

^j^ Chronic conditions include hypertension, diabetes, coronary heart disease, stroke, heart failure, arthritis, cancer, depression, dementia, Parkinson disease, and chronic obstructive pulmonary disease.

**Table 4.** Prospective Association of Factors With Sedentary Behavior and Physical Activity Among Women at 20 Years and 9 Years^a^

Factor	20-y Follow-up (n = 985)				9-y Follow-up (n = 954)			
	Mean time difference (95% CI), min/d^b^			*P* value for activity distribution^c^	Mean time difference (95% CI), min/d^b^			*P* value for activity distribution^c^
	SB	LIPA	MVPA		SB	LIPA	MVPA	
Sociodemographic factor								
Age, per 5 y	16.6 (11.4 to 21.9)	−6.0 (−10.1 to −1.8)	−10.7 (−12.6 to −8.7)	<.001^d^	18.9 (13.5 to 24.4)	−7.5 (−11.8 to −3.2)	−11.5 (−13.5 to −9.4)	<.001^d^
Member of racial or ethnic minority group^e^	9.2 (−10.3 to 28.7)	−4.8 (−20.0 to 10.5)	−4.4 (−11.6 to 2.8)	<.001^d^	8.5 (−12.0 to 28.9)	−3.7 (−19.8 to 12.4)	−4.8 (−12.4 to 2.8)	<.001^d^
Lower occupational position^f^	−9.3 (−27.7 to 9.2)	11.3 (−3.1 to 25.7)	−2.0 (−8.8 to 4.8)	.17	−17.4 (−36.2 to 1.3)	14.1 (−0.7 to 28.8)	3.4 (−3.6 to 10.3)	<.001^d^
Not married or cohabitating	15.0 (2.9 to 27.1)	−13.8 (−23.3 to −4.3)	−1.2 (−5.7 to 3.2)	<.001^d^	9.2 (−2.7 to 21.2)	−8.6 (−18.0 to 0.8)	−0.6 (−5.1 to 3.8)	<.001^d^
Behavioral factor								
Smoking status								
Never	1 [Reference]	1 [Reference]	1 [Reference]	NA	1 [Reference]	1 [Reference]	1 [Reference]	NA
Past	−4.7 (−18.1 to 8.6)	1.8 (−8.6 to 12.2)	3.0 (−2.0 to 7.9)	.84	−0.3 (−12.9 to 12.3)	−0.8 (−10.7 to 9.2)	1.1 (−3.6 to 5.8)	.33
Current	11.6 (−7.5 to 30.7)	−4.5 (−19.4 to 10.4)	−7.1 (−14.1 to −0.1)	.002^d^	10.0 (−12.4 to 32.3)	0.1 (−17.5 to 17.7)	−10.0 (−18.4 to −1.7)	<.001^d^
Alcohol intake^g^								
None	14.1 (−0.2 to 28.3)	−11.1 (−22.2 to 0.0)	−3.0 (−8.2 to 2.3)	<.001^d^	16.5 (1.9 to 31.2)	−12.6 (−24.1 to −1.1)	−4.0 (−9.4 to 1.5)	<.001^d^
Moderate	1 [Reference]	1 [Reference]	1 [Reference]	NA	1 [Reference]	1 [Reference]	1 [Reference]	NA
High	0.9 (−18.8 to 20.6)	−2.4 (−17.8 to 13.0)	1.5 (−5.8 to 8.7)	.15	1.3 (−16.0 to 18.6)	−5.6 (−19.2 to 7.9)	4.3 (−2.1 to 10.8)	<.001^d^
Fruit and vegetable intake < twice daily	7.8 (−5.2 to 20.8)	−6.3 (−16.5 to 3.9)	−1.5 (−6.3 to 3.3)	<.001^d^	9.8 (−2.7 to 22.4)	−8.3 (−18.2 to 1.5)	−1.5 (−6.2 to 3.2)	<.001^d^
Health-related factor								
BMI^h^								
Reference range	1 [Reference]	1 [Reference]	1 [Reference]	NA	1 [Reference]	1 [Reference]	1 [Reference]	NA
Overweight	34.8 (21.5 to 48.1)	−20.4 (−30.8 to −10.0)	−14.4 (−19.3 to −9.5)	<.001^d^	22.9 (9.4 to 36.5)	−11.1 (−21.8 to −0.4)	−11.9 (−16.9 to −6.8)	<.001^d^
Obese	44.8 (26.1 to 63.6)	−27.8 (−42.5 to −13.2)	−17.0 (−23.9 to −10.1)	<.001^d^	45.1 (29.8 to 60.5)	−27.3 (−39.4 to −15.2)	−17.9 (−23.6 to −12.1)	<.001^d^
MCS, per 10-point decrease^i^	8.4 (2.0 to 14.8)	−4.9 (−9.9 to 0.1)	−3.5 (−5.8 to −1.1)	<.001^d^	7.5 (1.6 to 13.5)	−5.1 (−9.8 to −0.4)	−2.4 (−4.7 to −0.2)	<.001^d^
PCS, per 10-point decrease^i^	6.7 (−0.9 to 14.2)	−1.8 (−7.6 to 4.1)	−4.9 (−7.7 to −2.1)	<.001^d^	7.4 (1.0 to 13.7)	−2.9 (−7.9 to 2.0)	−4.4 (−6.8 to −2.1)	<.001^d^
No. of chronic conditions, per new condition^j^	8.4 (−5.6 to 22.4)	−6.2 (−17.2 to 4.7)	−2.1 (−7.3 to 3.0)	.10	11.4 (3.7 to 19.0)	−8.8 (−14.9 to −2.8)	−2.5 (−5.4 to 0.3)	<.001^d^

Abbreviations: BMI, body mass index (calculated as weight in kilograms divided by height in meters squared); LIPA, light-intensity physical activity; MCS, mental component summary score from the 36-Item Short Form Health Survey; MVPA, moderate to vigorous physical activity; NA, not applicable; PCS, physical component summary score from the 36-Item Short Form Health Survey; SB, sedentary behavior.

^a^ Associations are between factors as measured in 1991 to 1993 (for 20 years of follow-up) or 2002 to 2004 (for 9 years of follow-up) and outcomes measured in 2012 to 2013.

^b^ Estimated from linear regression adjusted for all covariates and waking time.

^c^ *P* for association between each factor and activity intensity distribution from function-on-scalar regression adjusted for covariates and waking time.

^d^ *P* values < .004 were considered significant according to the Bonferroni correction for multiple testing.

^e^ Race and ethnicity were assessed by questionnaire using the response categories Black, South Asian, White, and other. Racial and ethnic minority groups have been combined in analysis owing to small numbers.

^f^ Last occupational position was defined as low (clerical or support positions), intermediate (professional or executive positions), or high (administrative positions), coded as 1, 0.5, and 0, respectively, and entered as an ordinal variable in analyses.

^g^ Alcohol intake was defined as 0 units/wk for none, 1 to 14 units/wk for moderate, and more than 14 units/wk for high.

^h^ BMI categories were defined as less than 25 for reference range, 25 to 29.9 for overweight, and 30 or more for obese.

^i^ Lower MCS and PCS correspond to poorer mental and physical summary score, respectively.

^j^ Chronic conditions include hypertension, diabetes, coronary heart disease, stroke, heart failure, arthritis, cancer, depression, dementia, Parkinson disease, and chronic obstructive pulmonary disease.

In the first sensitivity analysis, prospective models were additionally adjusted for self-reported time in MVPA at corresponding time points (eTables 3 and 4 in the Supplement), and results were similar to those of the main prospective analyses (Table 3 and Table 4). Participants reporting no MVPA in 1991 to 1993 spent more time in sedentary behavior (mean time difference, 26.8 min/d [95% CI, 14.8 to 38.8 min/d] in men) and less time in LIPA (mean time difference, −17.9 min/d [95% CI, −26.8 to −8.9 min/d] in men) and MVPA (−9.0 min/d [95% CI, −13.9 to −4.1 min/d] in men) in 2012 to 2013 compared with those reporting at least the recommended 2.5 h/wk of MVPA (*P* < .001) (eTable 3 in the Supplement). Findings were similar for analyses with self-reported MVPA and exposure variables assessed in 2002 to 2004. In the second sensitivity analysis, associations remained substantially the same when accounting for missing data using multiple imputation (eTables 5 and 6 in the Supplement).

## Discussion

In this cohort study, a large set of sociodemographic, behavioral, and health-related factors were found to be associated with accelerometer-assessed physical activity and sedentary behavior in older adults. The pattern of association was consistent whether these factors were assessed in midlife or later life. Factors associated with physical activity and sedentary behavior were overall similar in men and women, but the mean time differences were generally smaller among women. Taken together, these findings suggest the complexity of individual-level barriers associated with decreased activity in later life, with these barriers being already evident in midlife.

With a few exceptions,^20,21,22,23,30^ most data on factors associated with physical activity and sedentary behavior come from studies that examined factors associated with total physical activity, sedentary behavior, or MVPA separately. Three notable studies^21,22,23^ described the whole activity intensity distribution in older adults. Similar to our findings, men were found to spend more time in low and high acceleration ranges, while women spent more time in the middle acceleration range.^21,23^ This corroborates findings from studies considering time in different activity levels separately finding that men spent more time sedentary^17,24^ and in MVPA^14,22^ than women, who undertook more LIPA.^30^ Accounting for the full activity intensity distribution and time in different activity levels allowed us to gain a comprehensive understanding of time reallocation among subgroups of factors. For example, in men, a 5-year increase in age was associated with 15 more minutes per day in sedentary behavior, mainly at the expense of less time in MVPA, while male and female current smokers spent more time sedentary, but this increase was mainly at the expense of decreased time in LIPA for men and MVPA for women. These findings suggest the importance of considering the full activity intensity spectrum when examining its associated factors.

Previous studies on factors associated with physical activity or sedentary behavior in older age have mainly focused on age, sex, socioeconomic markers, smoking, BMI, and self-reported health, with health often assessed using a single-item question. In these studies, factors most consistently associated with decreased total physical activity were older age,^14,17,18,21,23,24,30^ increased BMI,^14,17,18,20,21,22,23,24,30^ and poorer self-reported health.^14,15,20,21,22^ Some studies have also reported an association of poor physical functioning^16,23^ or chronic conditions^15,18,19,23^ with total physical activity or time in activity levels. Our study used a comprehensive approach to assess the independent association of sociodemographic, behavioral, and health-related factors with physical activity and sedentary behavior in older age. Our findings are consistent with those of previous studies showing large differences in activity levels between obese and reference range weight groups, as well as differences by age and health status. Our results also suggest clustering of lifestyles, particularly evident in cross-sectional analyses, with individuals who were less sedentary and more physically active drinking more alcohol, eating more fruits and vegetables, and not smoking, independent of sociodemographic and health-related factors, as previously found in studies using self-reported physical activity measures.^31^ The observation that individuals who drink more alcohol were more physically active may reflect increased social interactions in this group.^32^ We also accounted for multimorbidity, using number of chronic conditions, and mental and physical health, which are important health dimensions in older adults^3^; both were found to be independently associated with different activity levels.

Most previous studies on factors associated with physical activity and sedentary behavior in older adults were cross-sectional.^11^ Notable prospective studies^18,19,20,22,24,30^ mainly focused on a specific rather than a holistic list of factors. Some of these studies, like ours, found an association between being more physically active earlier in life and at older age,^18,20,22^ suggesting that in midlife, individuals are already in distinct tracks of physical activity that remain over the life course. Obesity in midlife has also been reported to be consistently associated with decreased physical activity^18,20,30^ and increased sedentary time^19,24^ in older age. Our study using repeated data on a large set of sociodemographic, behavioral, and health-related factors adds to these results in finding these factors to be consistently associated with physical activity and sedentary behavior in older adults over a 20-year period.

Strengths of this study include its large sample size, use of objectively assessed physical activity and sedentary behavior, repeated measures of individual factors for investigation of prospective and cross-sectional associations, and consideration of activity intensity distribution and time in different activity levels. Use of function-on-scalar regression to examine factors associated with the whole activity intensity distribution allowed us to capture associations along the acceleration continuum. Although some thresholds have been proposed to define activity levels for wrist-worn accelerometers,^28^ so far there is no criterion standard, particularly in older adults. Using the continuum of acceleration allowed us to go beyond this limitation and consider the continuum of energy expenditure, without loss of information that could arise from categorizing activity levels. Advantages of this approach were evident in analyses in women in which function-on-scalar regressions identified more factors associated with activity intensity distribution than separate linear regression models for time in sedentary behavior, LIPA, and MVPA.

### Limitations

This study has several limitations. The Whitehall II study was originally an occupational cohort so participants were healthier than the general population. However, this may not have influenced findings of the association between factors and outcomes of interest; a previous study found that in the associations between several factors, including physical activity, and risk of incident cardiovascular disease from this cohort, outcomes were similar to those reported in general population-based studies.^33^ The proportion of women in our sample was small, meaning that the linear regression analyses conducted in women had lower statistical power than analyses in men in this study. Nonetheless, use of functional regression allowed us to investigate associations in women. Additionally, accelerometer data were assessed once, so we could not examine factors associated with changes in physical activity and sedentary behavior. Sensitivity analyses found that factors prospectively associated with physical activity and sedentary behavior in older age were similar, whether models controlled for previous self-reported physical activity or not.

## Conclusions

These findings may have important implications for public health strategies. Given that the population is aging, early detection of individuals likely to be inactive in later life may be useful in preventing detrimental outcomes associated with lack of physical activity and increased sedentary time, including onset of chronic conditions and functional limitations in older age. Current recommendations to reduce inactivity in older age are targeted toward the entire population and are often limited to a single behavioral factor. Results from this study suggest that prevalent overweight, obesity, and chronic conditions, as well as poor mental and physical health, could be used to screen individuals for inclusion in tailored interventions. Given the clustering of behavioral factors, such interventions may promote healthier lifestyles overall, rather than targeting a single behavior. This study also suggests the importance of implementing such intervention and policy strategies as early as possible given the consistency of factors associated with physical activity and sedentary behavior in older age over time.

## Notes

Supplement. **eMethods 1.** Procedure for Function-on-Scalar Regression **eMethods 2.** Exposure Variables: Sociodemographic, Behavioral, and Health-Related Factors **eFigure 1.** Study Flowchart **eFigure 2.** Cross-Sectional Association of Sociodemographic Factors With Activity Intensity Distribution **eFigure 3.** Cross-Sectional Association of Behavioral Factors With Activity Intensity Distribution **eFigure 4.** Cross-Sectional Association of Health-Related Factors With Activity Intensity Distribution **eTable 1.** Interactions of Sex With Factors for Association With Activity Intensity Distribution **eTable 2.** Characteristics of Participants Included and Not Included in Analyses **eTable 3.** Association of Exposure Variables With Sedentary Behavior and Physical Activity Among Men at 20 and 9 Years **eTable 4.** Association of Exposure Variables With Sedentary Behavior and Physical Activity Among Women at 20 and 9 Years **eTable 5.** Association of Exposure Factors With Outcomes Among 3552 Men Accounting for Missing Data Using Multiple Imputation **eTable 6.** Association of Exposure Factors With Outcomes Among 1328 Women Accounting for Missing Data Using Multiple Imputation
