# Supplementary material for: Individual Barriers to an Active Lifestyle at Older Ages Among Whitehall II Study Participants After 20 Years of Follow-up
Source: JAMA Netw Open. 2022 Apr 7;5(4):e226379. doi: 10.1001/jamanetworkopen.2022.6379 (PMC8990327; doi:10.1001/jamanetworkopen.2022.6379)
Supplement: Supplement. — eMethods 1. Procedure for Function-on-Scalar Regression eMethods 2. Exposure Variables: Sociodemographic, Behavioral, and Health-Related Factors eFigure 1. Study Flowchart eFigure 2. Cross-Sectional Association of Sociodemographic Factors With Activity Intensity Distribution eFigure 3. Cross-Sectional Association of Behavioral Factors With Activity Intensity Distribution eFigure 4. Cross-Sectional Association of Health-Related Factors With Activity Intensity Distribution eTable 1. Interactions of Sex With Factors for Association With Activity Intensity Distribution eTable 2. Characteristics of Participants Included and Not Included in Analyses eTable 3. Association of Exposure Variables With Sedentary Behavior and Physical Activity Among Men at 20 and 9 Years eTable 4. Association of Exposure Variables With Sedentary Behavior and Physical Activity Among Women at 20 and 9 Years eTable 5. Association of Exposure Factors With Outcomes Among 3552 Men Accounting for Missing Data Using Multiple Imputation eTable 6. Association of Exposure Factors With Outcomes Among 1328 Women Accounting for Missing Data Using Multiple Imputation [file jamanetwopen-e226379-s001.pdf]

Supplemental Online Content

Chen M, Yerramalla MS, van Hees VT, et al. Individual barriers to an active lifestyle at older ages among Whitehall II study participants after 20 years of follow-up. *JAMA Netw Open.* 2022;5(4):e226379. doi:10.1001/jamanetworkopen.2022.6379

eMethods 1. Procedure for Function-on-Scalar Regression

eMethods 2. Exposure Variables: Sociodemographic, Behavioral, and Health-Related Factors

eFigure 1. Study Flowchart

eFigure 2. Cross-Sectional Association of Sociodemographic Factors With Activity Intensity Distribution

eFigure 3. Cross-Sectional Association of Behavioral Factors With Activity Intensity Distribution

eFigure 4. Cross-Sectional Association of Health-Related Factors With Activity Intensity Distribution

eTable 1. Interactions of Sex With Factors for Association With Activity Intensity Distribution

eTable 2. Characteristics of Participants Included and Not Included in Analyses

eTable 3. Association of Exposure Variables With Sedentary Behavior and Physical Activity Among Men at 20 and 9 Years

eTable 4. Association of Exposure Variables With Sedentary Behavior and Physical Activity Among Women at 20 and 9 Years

eTable 5. Association of Exposure Factors With Outcomes Among 3552 Men Accounting for Missing Data Using Multiple Imputation

eTable 6. Association of Exposure Factors With Outcomes Among 1328 Women Accounting for Missing Data Using Multiple Imputation

This supplemental material has been provided by the authors to give readers additional information about their work.

© 2022 Chen M et al. *JAMA Network Open.*

eMethods 1. Procedure for Function-on-Scalar Regression

Estimation of activity intensity distribution

For each participant, the density function of acceleration during waking period was estimated by kernel density estimation on a logarithmically distributed 150-point grid, using a Gaussian kernel and a plug-in bandwidth selector. As acceleration distribution was skewed, the log transformed (1 + acceleration) was used for the kernel smoothing. The complete diurnal activity intensity distribution function was defined as follows:

$$A_i(x) = T_i * f_i(x)$$

where T_i and f_i are the daily waking time and the density function for the ith participant, respectively, x is the magnitude of acceleration variable which takes its values over the range of the recorded acceleration, and $A_i(x)$ represents the time spent at magnitude x. The integral of A_i over any range of acceleration represents the time (minutes) spent in this acceleration range per day, with integral over the total range of acceleration representing the daily waking time.

Function-on-scalar regression

Equation of the functional model adjusted for all covariates:

$$A_i(x) = \alpha(x) + \beta_{age}(x) * age_i + \beta_{sex}(x) * sex_i + \beta_{ethnicity}(x) * ethnicity_i + \beta_{position}(x) * position_i + \beta_{smok\_ex}(x) * Dsmok\_ex_i + \beta_{smok\_cur}(x) * Dsmok\_cur_i + \beta_{alc\_0}(x) * Dalc\_0_i + \beta_{alc\_14}(x) * Dalc\_14_i + \beta_{low\_fruit\_veg}(x) * low\_fruit\_veg_i + \beta_{bmi\_overweight}(x) * Dbmi\_overweight_i + \beta_{bmi\_obese}(x) * Dbmi\_obese_i + \beta_{mental\_score}(x) * mental\_score_i + \beta_{physical\_score}(x) * physical\_score_i + \beta_{multimorbidity\_index}(x) * multimorbidity\_index_i$$

In this model, $A_i(x)$ is the activity intensity distribution function for the ith participant and corresponds to the outcome. $\alpha(x)$ is the intercept activity distribution function. $\beta_{age}(x)$, $\beta_{sex}(x)$, $\beta_{ethnicity}(x)$, $\beta_{position}(x)$, $\beta_{smok\_ex}(x)$, $\beta_{smok\_cur}(x)$, $\beta_{alc\_0}(x)$, $\beta_{alc\_14}(x)$, $\beta_{low\_fruit\_veg}(x)$, $\beta_{bmi\_overweight}(x)$, $\beta_{bmi\_obese}(x)$, $\beta_{mental\_score}(x)$, $\beta_{physical\_score}(x)$, and $\beta_{multimorbidity\_index}(x)$ are the functional coefficients of the model; they represent the association between the covariates and the outcome. $Dsmok\_ex_i$, $Dsmok\_cur_i$, $Dalc\_0_i$, $Dalc\_14_i$, $Dbmi\_overweight_i$, and $Dbmi\_obese_i$ are "dummy" binary variables indicating the belonging to ex-smokers, current smokers, 0 unit per week alcohol intake, >14 units per week alcohol intake, overweight, and obesity groups, respectively.

In order to facilitate interpretation of results, functional regression coefficients were integrated over 0.005 *g* intervals to estimate the time difference (in minutes/day) in each acceleration interval between the different subgroups.

Software and packages

All analyses were undertaken using R version 3.6.1 (version 3.6.1, http://www.r-project.org/). Kernel smoothing was implemented using the package *ks* (version 1.11.7, https://cran.r-project.org/web/packages/ks/ks.pdf) of R. All function-on-scalar regressions were conducted using the *REFUND* package in R (version 0.1-21, https://cran.r-project.org/web/packages/refund/refund.pdf). The trapezoidal integration of functional coefficients was undertaken using the *pracma* R package (version 2.2.9, https://cran.r-project.org/web/packages/pracma/pracma.pdf). Significance of associations was examined using p-values from function-on-scalar regression, that accounts for the entire activity intensity distribution, after Bonferroni correction leading to p<0.004 (0.05 divided by 14 tests per model).

eMethods 2. Exposure Variables: Sociodemographic, Behavioral, and Health-Related Factors

Data were drawn from questionnaires and clinical evaluations as well as from electronic health records (Hospital Episode Statistics (HES), cancer registry, and the Mental Health Services Data Set).

Socio-demographic factors included age, sex, self-reported ethnicity (categorized as white and non-white (Black; South Asian; other), due to small numbers of non-white groups), marital status (married/cohabitating, divorced/widowed/single), and last occupational position (low, intermediate, high; coded 1, 0.5, and 0, respectively, and entered as an ordinal variable; this is a comprehensive marker of socioeconomic position in the British Civil Service that reflects salary, social status, and level of responsibility at work).

Behavioral factors were smoking status (never, past, current smoker), alcohol intake (none, moderate (1-14 units/week), high (>14 units/week)), and fruit and vegetable intake (<twice daily, ≥twice daily).

Health-related factors included body mass index (BMI; categorized as <25 (normal), 25-29.9 (overweight), and ≥30 (obese) kg/m^2), Short Form 36 Health Survey[1] physical and mental component summary scores, and number of chronic conditions including hypertension (systolic/diastolic blood pressure ≥140/90 mmHg, or use of antihypertensive drugs), diabetes (fasting glucose ≥7.0 mmol/L, self-reported doctor-diagnosed diabetes, use of anti-diabetic medications, or record in HES), coronary heart disease, stroke, heart failure, arthritis, cancer, depression, dementia, Parkinson's disease, and chronic obstructive pulmonary disease (assessed using HES records and data collected at Whitehall clinical exams as well as mental health records for depression and dementia).

Reference:

1. Ware JE, Jr., Kosinski M, Bayliss MS, et al. Comparison of methods for the scoring and statistical analysis of SF-36 health profile and summary measures: summary of results from the Medical Outcomes Study. *Medical care* 1995;33(4 Suppl):As264-79. [published Online First: 1995/04/01]

© 2022 Chen M et al. *JAMA Network Open.*

eFigure 1. Study Flowchart

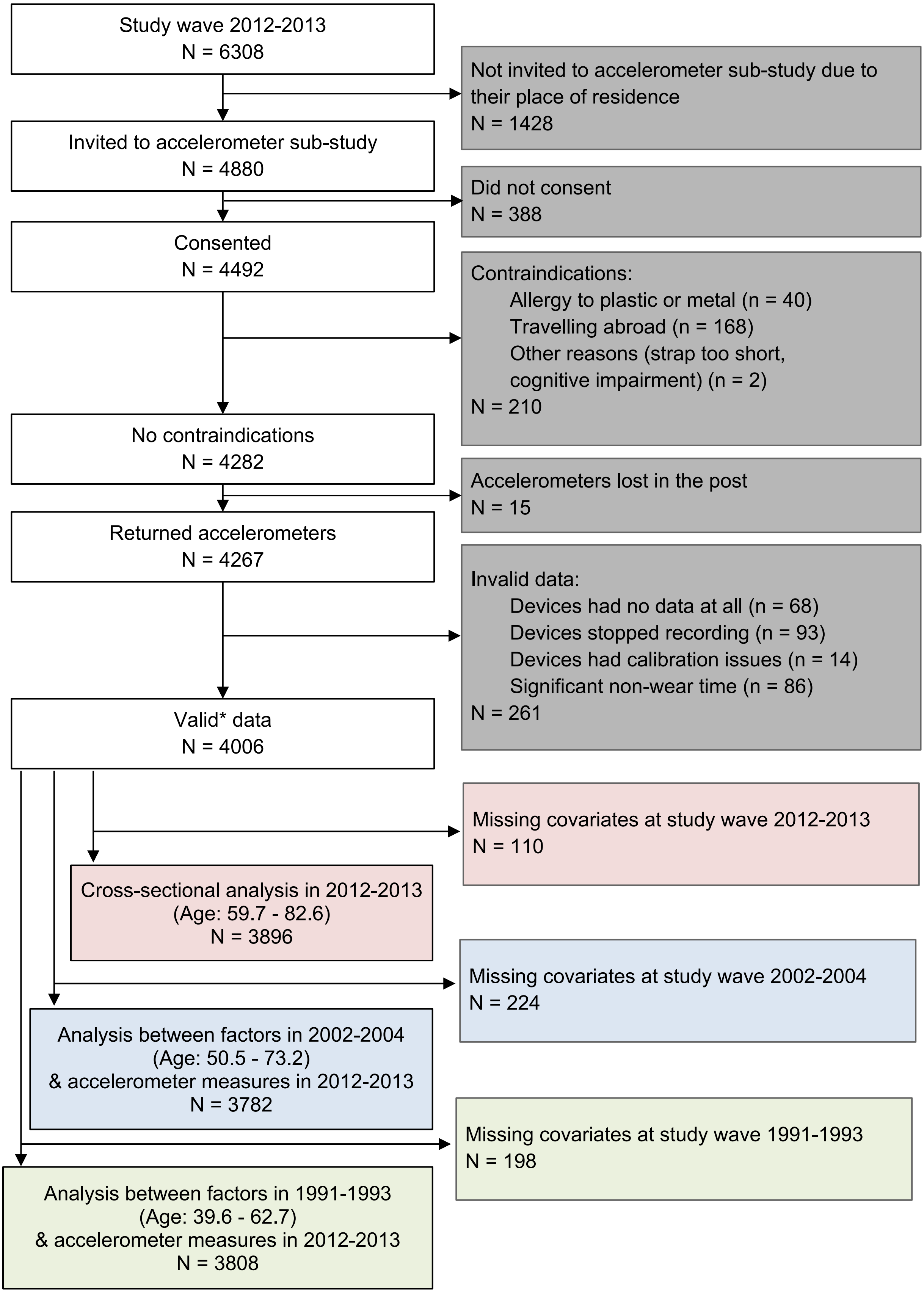

In total, 3809 (98%) participants had 7 days of valid data, 47 (1%) had 6 days, 22 (<1%) had 5 days, and 18 (<1%), had 4 days of valid data.

© 2022 Chen M et al. *JAMA Network Open.*

eFigure 2. Cross-Sectional Association of Sociodemographic Factors With Activity Intensity Distribution

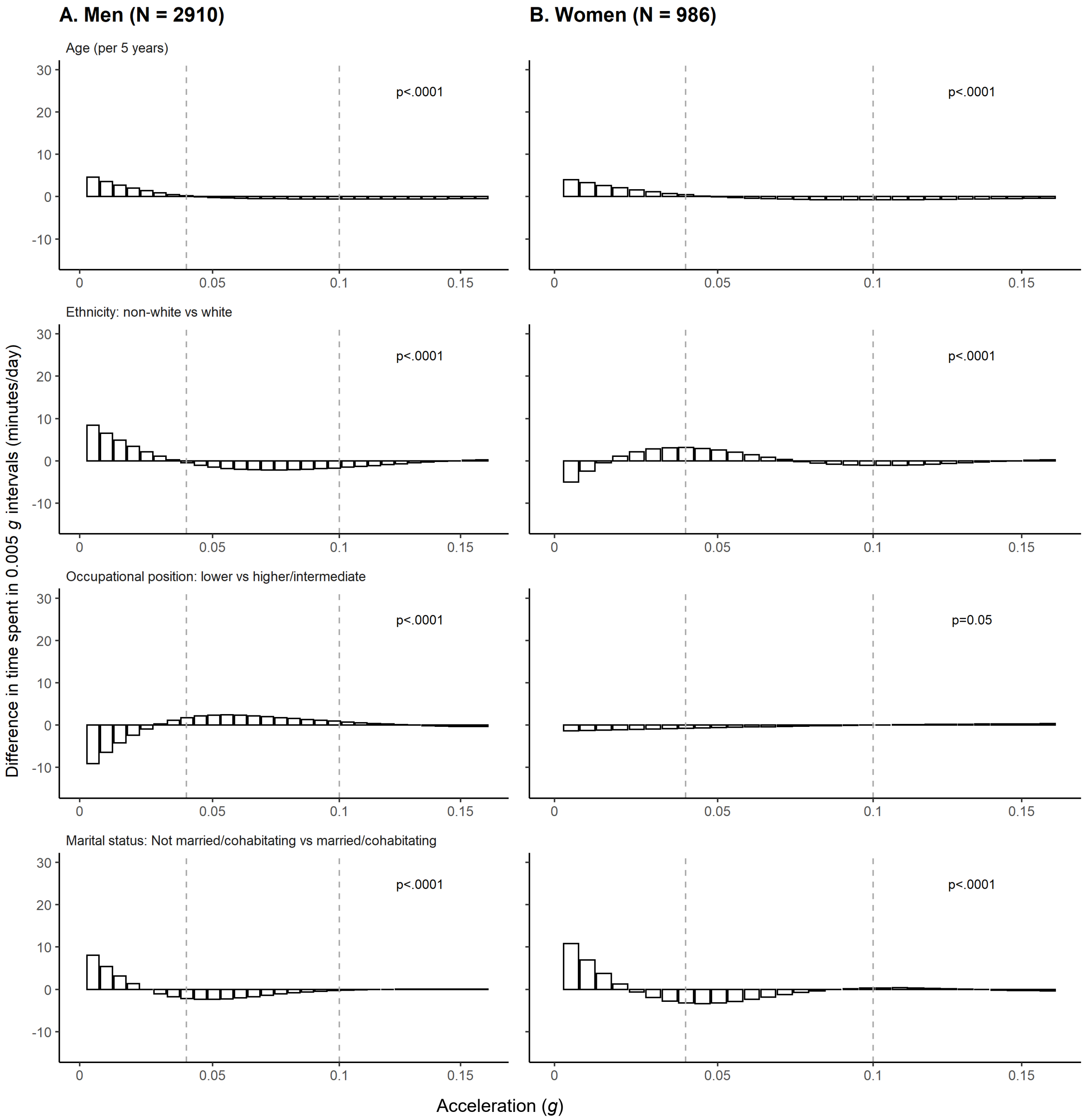

Time difference (minutes/day) spent over 0.005 *g* acceleration intervals between different subgroups, computed after integration of activity intensity distribution estimated by function-on-scalar regression model adjusted for all covariates (sociodemographic, behavioral, and health-related factors) and waking time. *p* for association between each covariate and the full activity intensity distribution from function-on-scalar regression mutually adjusted for all covariates and waking time.

© 2022 Chen M et al. *JAMA Network Open.*

eFigure 3. Cross-Sectional Association of Behavioral Factors With Activity Intensity Distribution

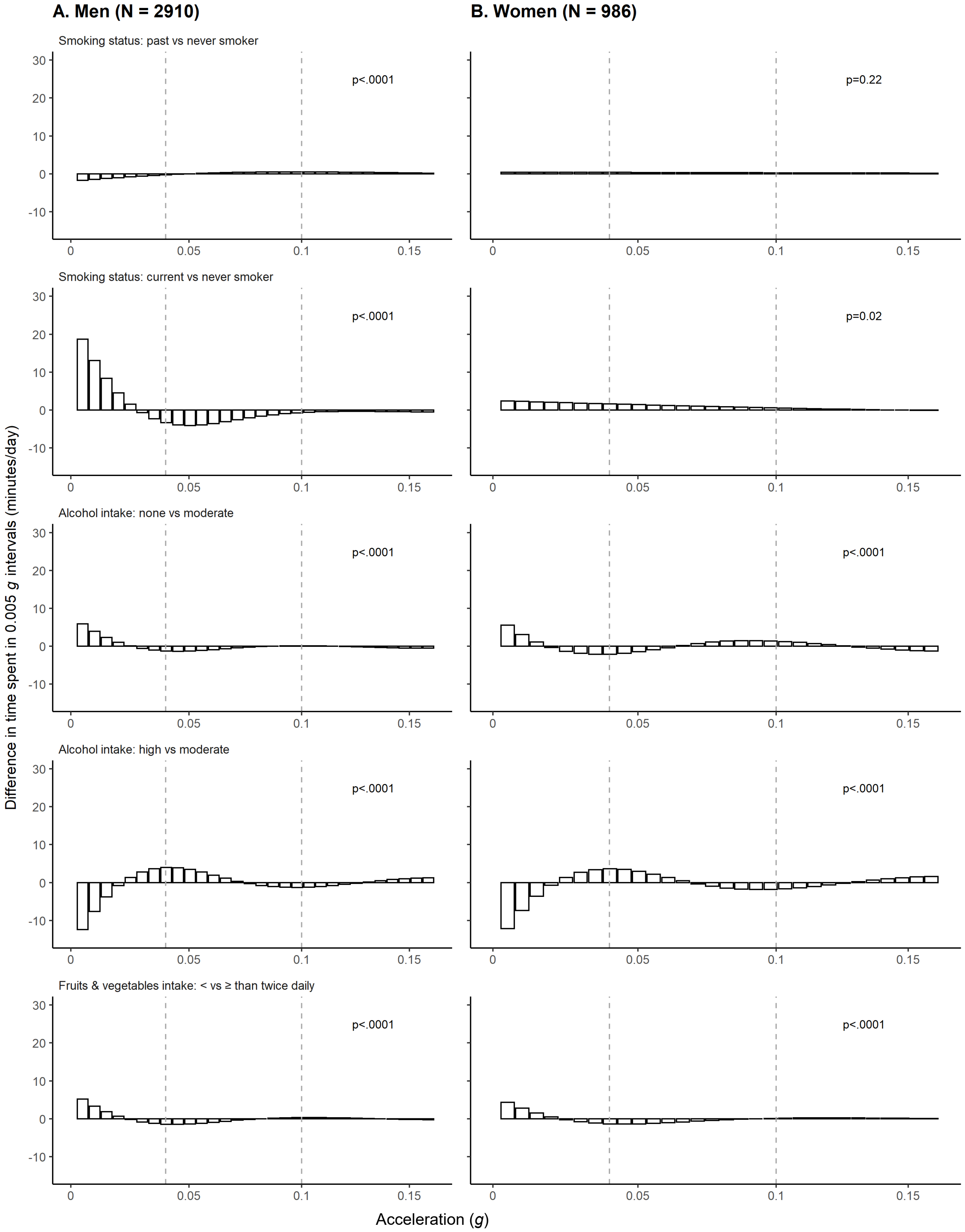

Time difference (minutes/day) spent over 0.005 *g* acceleration intervals between different subgroups, computed after integration of activity intensity distribution estimated by function-on-scalar regression model adjusted for all covariates (sociodemographic, behavioral, and health-related factors) and waking time. *p* for association between each covariate and the full activity intensity distribution from function-on-scalar regression mutually adjusted for all covariates and waking time.

© 2022 Chen M et al. *JAMA Network Open.*

eFigure 4. Cross-Sectional Association of Health-Related Factors With Activity Intensity Distribution

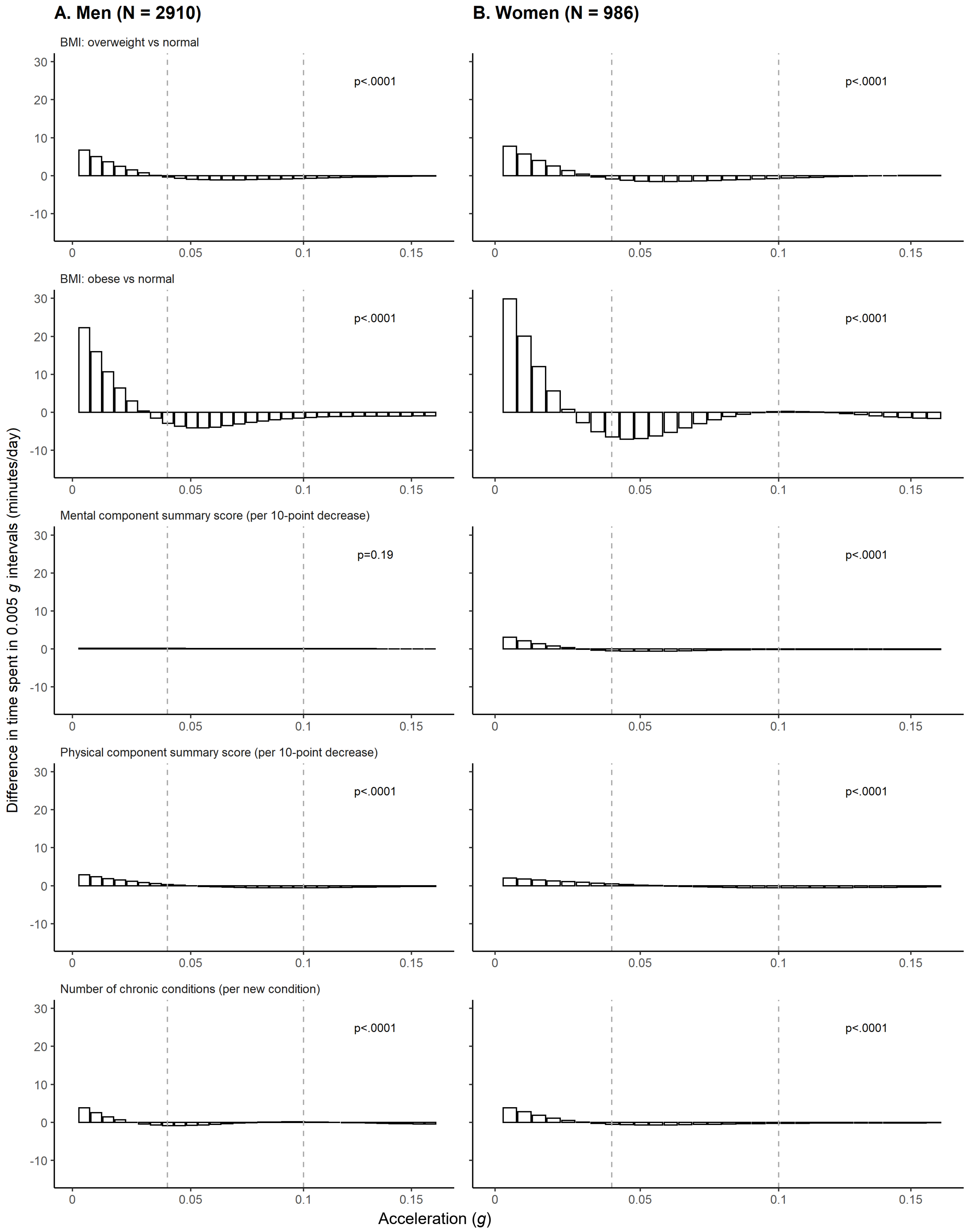

Time difference (minutes/day) spent over 0.005 *g* acceleration intervals between different subgroups, computed after integration of activity intensity distribution estimated by function-on-scalar regression model adjusted for all covariates (sociodemographic, behavioral, and health-related factors) and waking time. *p* for association between each covariate and the full activity intensity distribution from function-on-scalar regression mutually adjusted for all covariates and waking time.

© 2022 Chen M et al. *JAMA Network Open.*

eTable 1. Interactions of Sex With Factors for Association With Activity Intensity Distribution

	1991-1993 (N=3808) [a]	2002-2004 (N=3782) [a]	2012-2013 (N=3896) [a]
**Socio-demographic factors**			
Age	0.057	0.91	0.69
Non-white ethnicity	<.001	<.001	<.001
Lower occupational position	<.001	<.001	<.001
Not married/cohabitating	0.00012	0.79	0.03
**Behavioral factors**			
Smoking status			
Never smoker	Ref	Ref	Ref
Past smoker	<.001	<.001	<.001
Current smoker	<.001	<.001	<.001
Alcohol intake			
None	0.0025	<.001	0.27
Moderate	Ref	Ref	Ref
High	0.99	0.61	0.97
Fruits & vegetables intake < twice daily	0.99	0.93	0.58
**Health-related factors**			
Body mass index			
Normal	Ref	Ref	Ref
Overweight	<.001	<.001	0.85
Obese	0.67	0.64	<.001
SF36-mental component summary score	0.22	0.0012	<.001
SF36-physical component summary score	0.78	0.28	0.33
Number of chronic conditions [b]	<.001	<.001	0.66

Abbreviations: SF36: Short Form 36 Health Survey.
[a] *p* from function-on-scalar regression assessing interactions of sex with covariates.
[b] Chronic conditions include hypertension, diabetes, coronary heart disease, stroke, heart failure, arthritis, cancer, depression, dementia, Parkinson's disease, and chronic obstructive pulmonary disease.

eTable 2. Characteristics of Participants Included and Not Included in Analyses

	1991-1993				2002-2004				2012-2013		
	**Included N=3808**	**Not included N=1072**	***p*** [a]		**Included N=3782**	**Not included N=1098**	***p*** [a]		**Included N=3896**	**Not included N=984**	***p*** [a]
**Socio-demographic factors**											
Age, mean (SD)	49.1 (5.7)	48.6 (5.7)	0.03		60.4 (5.7)	59.5 (5.6)	<.001		69.4 (5.7)	68.9 (5.6)	0.009
Sex											
Men	2823 (74.1%)	729 (68.0%)	<.001		2828 (74.8%)	724 (65.9%)	<.001		2910 (74.7%)	642 (65.2%)	<.001
Women	985 (25.9%)	343 (32.0%)			954 (25.2%)	374 (34.1%)			986 (25.3%)	342 (34.8%)	
Ethnicity											
White	3532 (92.8%)	960 (89.6%)	<.001		3523 (93.2%)	969 (88.3%)	<.001		3622 (93.0%)	870 (88.4%)	<.001
Non-white	276 (7.2%)	112 (10.4%)			259 (6.8%)	129 (11.7%)			274 (7.0%)	114 (11.6%)	
Occupational position											
High/intermediate	3396 (89.2%)	933 (87.0%)	0.05		3458 (91.4%)	869 (88.1%)	0.001		3563 (91.5%)	870 (88.4%)	0.003
Low	412 (10.8%)	139 (13.0%)			324 (8.6%)	117 (11.9%)			333 (8.5%)	114 (11.6%)	
Missing	0	0			0	112			0	0	
Marital status											
Married/cohabitating	2978 (78.2%)	825 (77.0%)	0.41		2903 (76.8%)	726 (76.4%)	0.83		2916 (74.8%)	719 (73.5%)	0.39
Not married/cohabitating	830 (21.8%)	246 (23.0%)			879 (23.2%)	224 (23.6%)			980 (25.2%)	259 (26.5%)	
Missing	0	1			0	148			0	6	
**Behavioral factors**											
Smoking status											
Never smoker	2026 (53.2%)	549 (51.5%)	0.03		1903 (50.3%)	476 (50.7%)	0.61		1875 (48.1%)	420 (47.6%)	0.33
Past smoker	1406 (36.9%)	381 (35.8%)			1624 (42.9%)	392 (41.7%)			1897 (48.7%)	426 (48.2%)	
Current smoker	376 (9.9%)	135 (12.7%)			255 (6.7%)	71 (7.6%)			124 (3.2%)	37 (4.2%)	
Missing	0	7			0	159			0	101	
Alcohol intake											
None	612 (16.1%)	164 (15.3%)	0.32		548 (14.5%)	160 (17.1%)	0.04		777 (19.9%)	216 (22.1%)	0.31
Moderate	2238 (58.8%)	612 (57.2%)			2043 (54.0%)	466 (49.8%)			2207 (56.6%)	534 (54.6%)	
High	958 (25.2%)	293 (27.4%)			1191 (31.5%)	310 (33.1%)			912 (23.4%)	228 (23.3%)	

© 2022 Chen M et al. *JAMA Network Open.*

	1991-1993			2002-2004			2012-2013		
	**Included N=3808**	**Not included N=1072**	***p*** [a]	**Included N=3782**	**Not included N=1098**	***p*** [a]	**Included N=3896**	**Not included N=984**	***p*** [a]
Missing	0	3		0	162		0	6	
Fruits & vegetables intake									
< twice daily	861 (22.6%)	191 (23.0%)	0.79	1565 (41.4%)	384 (40.6%)	0.68	2268 (58.2%)	579 (59.4%)	0.51
≥ twice daily	2947 (77.4%)	638 (77.0%)		2217 (58.6%)	561 (59.4%)		1628 (41.8%)	396 (40.6%)	
Missing	0	243		0	153		0	9	
**Health-related factors**									
Body mass index									
Normal	2124 (55.8%)	619 (57.7%)	0.37	1449 (38.3%)	337 (37.9%)	0.67	1511 (38.8%)	380 (38.9%)	0.17
Overweight	1404 (36.9%)	370 (34.5%)		1712 (45.3%)	395 (44.4%)		1677 (43.0%)	396 (40.5%)	
Obese	280 (7.4%)	83 (7.7%)		621 (16.4%)	157 (17.7%)		708 (18.2%)	201 (20.6%)	
Missing	0	0		0	209		0	7	
SF36-mental component summary score									
Mean (SD)	51.0 (8.5)	50.9 (8.3)	0.83	52.2 (8.9)	51.4 (9.1)	0.02	53.8 (8.3)	53.4 (7.9)	0.24
Missing	0	247		0	183		0	33	
SF36-physical component summary score									
Mean (SD)	52.9 (6.5)	52.4 (7.2)	0.06	49.8 (8.2)	49.5 (8.4)	0.41	48.3 (8.9)	48.0 (9.1)	0.41
Missing	0	247		0	183		0	33	
Number of chronic conditions, mean (SD) [b]	0.2 (0.5)	0.2 (0.5)	0.63	0.7 (0.8)	0.6 (0.8)	0.005	1.2 (1.1)	1.2 (1.0)	0.325
**Accelerometer variables, time (min/day) in:**									
Sedentary behavior, mean (SD)							718.2 (100.0)	688.1 (152.2)	<.001
Light-intensity physical activity, mean (SD)							210.1 (69.1)	214.0 (81.9)	0.044
Moderate-to-vigorous physical activity, mean (SD)							56.1 (38.5)	74.1 (119.4)	<.001
Missing							0	787	

Data are No. (%) unless otherwise indicated. Abbreviations: SD, standard deviation; SF36: Short Form 36 Health Survey.

[a] *p* for difference between included and non-included participants at each time point.

[b] Chronic conditions include hypertension, diabetes, coronary heart disease, stroke, heart failure, arthritis, cancer, depression, dementia, Parkinson's disease, and chronic obstructive pulmonary disease.

© 2022 Chen M et al. *JAMA Network Open.*

eTable 3. Association of Exposure Variables With Sedentary Behavior and Physical Activity Among Men at 20 and 9 Years

	20-year follow-up (N=2823)				9-year follow-up (N=2828)			
	Time difference (95% CI) in: [a]			*p* [b] for activity distribution	Time difference (95% CI) in: [a]			*p* [b] for activity distribution
	SB	LIPA	MVPA		SB	LIPA	MVPA	
**Socio-demographic factors**								
Age (per 5 years)	17.0 (14.1, 19.9)	-6.1 (-8.2, -3.9)	-11.0 (-12.1, -9.8)	<.001*	18.5 (15.6, 21.5)	-7.0 (-9.2, -4.8)	-11.5 (-12.7, -10.3)	<.001*
Non-white ethnicity	25.8 (11.2, 40.4)	-19.0 (-29.9, -8.1)	-6.8 (-12.8, -0.9)	<.001*	22.3 (7.9, 36.6)	-16.4 (-27.2, -5.7)	-5.8 (-11.7, 0.0)	<.001*
Lower occupational position	-13.1 (-25.1, -1.1)	12.6 (3.7, 21.6)	0.4 (-4.4, 5.3)	<.001*	-20.8 (-32.7, -8.9)	17.4 (8.4, 26.3)	3.4 (-1.5, 8.2)	<.001*
Not married/cohabitating	10.1 (1.2, 19.0)	-12.3 (-19.0, -5.6)	2.2 (-1.4, 5.8)	<.001*	8.9 (0.2, 17.5)	-12.1 (-18.6, -5.6)	3.2 (-0.3, 6.8)	<.001*
**Behavioral factors**								
Smoking status								
Never smoker	Ref	Ref	Ref		Ref	Ref	Ref	
Past smoker	-7.7 (-14.6, -0.9)	5.6 (0.5, 10.7)	2.1 (-0.6, 4.9)	<.001*	-8.5 (-15.0, -1.9)	5.8 (0.9, 10.7)	2.7 (-0.0, 5.3)	<.001*
Current smoker	19.2 (7.7, 30.6)	-11.2 (-19.7, -2.6)	-8.0 (-12.7, -3.3)	<.001*	22.7 (9.4, 35.9)	-14.6 (-24.6, -4.6)	-8.1 (-13.5, -2.7)	<.001*
Alcohol intake								
None	4.2 (-5.7, 14.2)	-1.5 (-9.0, 5.9)	-2.7 (-6.8, 1.4)	<.001*	8.2 (-2.4, 18.8)	-5.5 (-13.4, 2.5)	-2.7 (-7.0, 1.6)	<.001*
Moderate	Ref	Ref	Ref		Ref	Ref	Ref	
High	2.1 (-5.2, 9.3)	-1.5 (-7.0, 3.9)	-0.5 (-3.5, 2.4)	<.001*	-3.2 (-10.0, 3.6)	3.2 (-1.9, 8.3)	-0.0 (-2.8, 2.8)	<.001*
Fruits & vegetables intake <twice daily	12.5 (4.6, 20.5)	-7.4 (-13.4, -1.5)	-5.1 (-8.3, -1.8)	<.001*	6.8 (0.4, 13.3)	-3.2 (-8.1, 1.6)	-3.6 (-6.2, -0.9)	<.001*
Self-reported MVPA								
None	26.8 (14.8, 38.8)	-17.9 (-26.8, -8.9)	-9.0 (-13.8, -4.1)	<.001*	45.4 (31.4, 59.4)	-35.5 (-46.0, -25.0)	-9.9 (-15.6, -4.1)	<.001*
0.1-2.4 h/week	15.9 (9.0, 22.7)	-8.7 (-13.8, -3.6)	-7.1 (-9.9, -4.3)	<.001*	17.2 (10.3, 24.1)	-11.1 (-16.2, -5.9)	-6.1 (-8.9, -3.3)	<.001*
≥2.5 h/week	Ref	Ref	Ref		Ref	Ref	Ref	
**Health-related factors**								
Body mass index								
Normal	Ref	Ref	Ref		Ref	Ref	Ref	
Overweight	16.8 (10.1, 23.5)	-8.3 (-13.4, -3.3)	-8.5 (-11.2, -5.7)	<.001*	13.1 (6.3, 19.9)	-5.6 (-10.8, -0.5)	-7.5 (-10.3, -4.7)	<.001*
Obese	50.0 (35.8, 64.2)	-27.0 (-37.6, -16.4)	-23.0 (-28.7, -17.2)	<.001*	44.7 (34.6, 54.7)	-24.6 (-32.2, -17.1)	-20.1 (-24.2, -16.0)	<.001*
MCS (per 10-point decrease) [c]	0.9 (-3.1, 5.0)	0.3 (-2.8, 3.3)	-1.2 (-2.9, 0.4)	<.001*	1.1 (-2.7, 5.0)	0.2 (-2.6, 3.1)	-1.4 (-2.9, 0.2)	0.94
PCS (per 10-point decrease) [c]	8.1 (2.3, 13.9)	-3.1 (-7.4, 1.2)	-5.0 (-7.3, -2.6)	<.001*	7.0 (2.6, 11.4)	-2.9 (-6.2, 0.4)	-4.1 (-5.9, -2.3)	<.001*
Number of chronic conditions [d] (per new condition)	12.7 (5.6, 19.8)	-7.6 (-12.9, -2.3)	-5.1 (-8.0, -2.2)	<.001*	13.5 (9.2, 17.8)	-8.0 (-11.3, -4.8)	-5.5 (-7.2, -3.7)	<.001*

Abbreviations: CI: confidence interval; SB: sedentary behavior; LIPA: light-intensity physical activity; MVPA: moderate-to-vigorous physical activity; MCS: Mental component summary score from the Short Form 36 Health Survey; PCS: SF 36 Physical component summary score from the Short Form 36 Health Survey.

[a] estimated from linear regression adjusted for all covariates (including self-reported MVPA) and waking time.

[b] *p* for association between covariates and activity intensity distribution from function-on-scalar regression mutually adjusted for all covariates and waking time; *p-values <0.004 were considered significant according to the Bonferroni correction for multiple testing.

[c] Lower MCS and PCS correspond to poorer mental and physical summary score, respectively.

[d] Chronic conditions include hypertension, diabetes, coronary heart disease, stroke, heart failure, arthritis, cancer, depression, dementia, Parkinson’s disease, and chronic obstructive pulmonary disease.

© 2022 Chen M et al. *JAMA Network Open.*

eTable 4. Association of Exposure Variables With Sedentary Behavior and Physical Activity Among Women at 20 and 9 Years

	20-year follow-up (N=985)				9-year follow-up (N=954)			
	Time difference (95% CI) in: [a]			*p* [b] for activity distribution	Time difference (95% CI) in: [a]			*p* [b] for activity distribution
	SB	LIPA	MVPA		SB	LIPA	MVPA	
**Socio-demographic factors**								
Age (per 5 years)	16.1 (10.8, 21.5)	-5.7 (-9.9, -1.6)	-10.4 (-12.4, -8.5)	<.001*	19.9 (14.5, 25.3)	-8.2 (-12.5, -4.0)	-11.6 (-13.6, -9.6)	<.001*
Non-white ethnicity	8.4 (-11.2, 28.0)	-4.4 (-19.7, 10.9)	-4.0 (-11.2, 3.2)	<.001*	6.3 (-14.0, 26.5)	-2.0 (-17.9, 14.0)	-4.3 (-11.9, 3.3)	<.001*
Lower occupational position	-7.2 (-25.6, 11.3)	10.0 (-4.5, 24.5)	-2.9 (-9.7, 3.9)	0.557	-16.0 (-34.6, 2.6)	13.1 (-1.6, 27.7)	2.9 (-4.0, 9.9)	<.001*
Not married/ cohabitating	15.5 (3.4, 27.5)	-14.1 (-23.5, -4.6)	-1.4 (-5.9, 3.1)	<.001*	9.1 (-2.7, 21.0)	-8.5 (-17.8, 0.8)	-0.6 (-5.1, 3.8)	<.001*
**Behavioral factors**								
Smoking status								
Never smoker	Ref	Ref	Ref					
Past smoker	-5.0 (-18.3, 8.2)	1.9 (-8.5, 12.3)	3.1 (-1.8, 8.0)	0.797	1.0 (-11.5, 13.5)	-1.8 (-11.6, 8.0)	0.9 (-3.8, 5.5)	0.054
Current smoker	11.6 (-7.4, 30.6)	-4.5 (-19.4, 10.4)	-7.1 (-14.1, -0.1)	0.001*	10.3 (-11.9, 32.5)	-0.4 (-17.9, 17.0)	-9.9 (-18.2, -1.5)	<.001*
Alcohol intake								
None	13.6 (-0.6, 27.8)	-10.9 (-22.0, 0.3)	-2.7 (-7.9, 2.5)	<.001*	14.3 (-0.3, 28.8)	-10.8 (-22.3, 0.6)	-3.4 (-8.9, 2.0)	<.001*
Moderate	Ref	Ref	Ref					
High	2.0 (-17.6, 21.6)	-3.0 (-18.4, 12.3)	1.0 (-6.2, 8.2)	0.294	0.6 (-16.5, 17.7)	-5.1 (-18.5, 8.4)	4.5 (-1.9, 10.9)	<.001*
Fruits & vegetables intake <twice daily	6.3 (-6.7, 19.3)	-5.4 (-15.6, 4.7)	-0.9 (-5.7, 3.9)	<.001*	6.9 (-5.6, 19.4)	-6.1 (-15.9, 3.8)	-0.8 (-5.5, 3.9)	<.001*
Self-reported MVPA								
None	17.1 (2.1, 32.2)	-9.6 (-21.4, 2.3)	-7.6 (-13.1, -2.0)	<.001*	38.7 (18.5, 58.8)	-33.1 (-48.9, -17.2)	-5.6 (-13.1, 2.0)	<.001*
0.1-2.4 h/week	19.6 (5.9, 33.2)	-11.6 (-22.3, -0.9)	-8.0 (-13.0, -3.0)	<.001*	21.8 (9.3, 34.2)	-15.9 (-25.7, -6.1)	-5.9 (-10.5, -1.2)	<.001*
≥2.5 h/week	Ref	Ref	Ref		Ref	Ref	Ref	
**Health-related factors**								
Body mass index								
Normal	Ref	Ref	Ref		Ref	Ref	Ref	
Overweight	33.8 (20.6, 47.1)	-19.9 (-30.2, -9.5)	-14.0 (-18.8, -9.1)	<.001*	22.0 (8.5, 35.4)	-10.4 (-21.0, 0.2)	-11.6 (-16.6, -6.5)	<.001*
Obese	43.2 (24.5, 61.9)	-26.9 (-41.5, -12.2)	-16.3 (-23.2, -9.5)	<.001*	43.6 (28.4, 58.9)	-26.1 (-38.1, -14.1)	-17.5 (-23.2, -11.8)	<.001*
MCS (per 10 points decrease) [c]	7.4 (1.0, 13.8)	-4.3 (-9.3, 0.7)	-3.1 (-5.4, -0.7)	<.001*	6.0 (0.1, 12.0)	-4.0 (-8.7, 0.7)	-2.0 (-4.3, 0.2)	<.001*
PCS (per 10 points decrease) [c]	5.5 (-2.0, 13.0)	-1.1 (-7.0, 4.8)	-4.4 (-7.2, -1.6)	<.001*	5.9 (-0.4, 12.2)	-1.8 (-6.7, 3.2)	-4.1 (-6.4, -1.7)	<.001*
Number of chronic conditions [d] (per new condition)	8.8 (-5.2, 22.7)	-6.5 (-17.4, 4.5)	-2.3 (-7.4, 2.8)	0.065	10.8 (3.2, 18.4)	-8.3 (-14.2, -2.3)	-2.5 (-5.4, 0.3)	<.001*

Abbreviations: CI: confidence interval; SB: sedentary behavior; LIPA: light-intensity physical activity; MVPA: moderate-to-vigorous physical activity; MCS: Mental component summary score from the Short Form 36 Health Survey; PCS: SF 36 Physical component summary score from the Short Form 36 Health Survey.

[a] estimated from linear regression adjusted for all covariates (including self-reported MVPA) and waking time.

[b] *p* for association between covariates and activity intensity distribution from function-on-scalar regression mutually adjusted for all covariates and waking time; *p-values <0.004 were considered significant according to the Bonferroni correction for multiple testing.

[c] Lower MCS and PCS correspond to poorer mental and physical summary score, respectively.

[d] Chronic conditions include hypertension, diabetes, coronary heart disease, stroke, heart failure, arthritis, cancer, depression, dementia, Parkinson's disease, and chronic obstructive pulmonary disease.

© 2022 Chen M et al. *JAMA Network Open.*

eTable 5. Association of Exposure Factors With Outcomes Among 3552 Men Accounting for Missing Data Using Multiple Imputation

	20-year follow-up			9-year follow-up			Cross-sectional analyses		
	Time difference (95% CI) in: [a]			Time difference (95% CI) in: [a]			Time difference (95% CI) in: [a]		
	SB	LIPA	MVPA	SB	LIPA	MVPA	SB	LIPA	MVPA
**Socio-demographic factors**									
Age (per 5 years)	19.1 (15.9, 22.3)	-7.3 (-9.6, -4.9)	-11.9 (-13.4, -10.3)	19.9 (16.6, 23.3)	-7.4 (-9.8, -4.9)	-12.5 (-14.1, -10.9)	17.4 (14.2, 20.7)	-6.0 (-8.4, -3.6)	-11.4 (-13.0, -9.9)
Non-white ethnicity	25.4 (11.9, 38.8)	-15.7 (-25.7, -5.6)	-9.7 (-16.1, -3.3)	21.2 (7.9, 34.5)	-12.5 (-22.5, -2.6)	-8.7 (-15.1, -2.3)	19.1 (6.1, 32.2)	-11.9 (-21.7, -2.1)	-7.3 (-13.5, -1.0)
Lower occupational position	-10.0 (-26.9, 6.8)	7.3 (-5.1, 19.6)	2.8 (-5.3, 10.8)	-11.5 (-29.3, 6.4)	7.1 (-6.3, 20.6)	4.3 (-3.9, 12.5)	-7.4 (-24.3, 9.5)	4.2 (-8.7, 17.1)	3.2 (-4.6, 10.9)
Not married/ cohabitating	12.3 (3.6, 21.1)	-14.0 (-20.3, -7.7)	1.6 (-2.6, 5.9)	10.0 (1.3, 18.6)	-13.2 (-19.5, -7.0)	3.3 (-0.9, 7.4)	9.6 (1.2, 17.8)	-13.0 (-18.9, -7.0)	3.4 (-0.6, 7.4)
**Behavioral factors**									
Smoking status									
Never smoker	Ref	Ref	Ref	Ref	Ref	Ref	Ref	Ref	Ref
Past smoker	-7.8 (-14.5, -1.0)	6.1 (1.1, 11.0)	1.7 (-1.6, 5.0)	-7.9 (-14.4, -1.4)	5.2 (0.4, 9.9)	2.7 (-0.5, 5.9)	-7.6 (-13.9, -1.3)	4.6 (0.0, 9.3)	2.9 (-0.3, 6.1)
Current smoker	18.3 (6.9, 29.6)	-9.8 (-18.0, -1.6)	-8.5 (-13.8, -3.1)	19.2 (6.1, 32.4)	-11.4 (-20.9, -1.8)	-7.9 (-14.1, -1.6)	30.0 (12.4, 47.7)	-17.0 (-30.0, -4.1)	-13.0 (-21.5, -4.5)
Alcohol intake									
None	4.5 (-5.3, 14.3)	-2.2 (-9.4, 4.9)	-2.3 (-7.0, 2.5)	6.6 (-4.1, 17.4)	-4.8 (-12.7, 3.1)	-1.8 (-7.1, 3.4)	8.1 (-0.9, 17.1)	-2.9 (-9.6, 3.8)	-5.2 (-9.5, -0.9)
Moderate	Ref	Ref	Ref	Ref	Ref	Ref	Ref	Ref	Ref
High	0.7 (-6.3, 7.6)	-0.4 (-5.6, 4.8)	-0.3 (-3.6, 3.0)	-6.8 (-13.7, 0.1)	4.8 (-0.3, 9.9)	2.0 (-1.3, 5.3)	-12.8 (-19.9, -5.7)	8.7 (3.6, 13.8)	4.1 (0.7, 7.5)
Fruits & vegetables intake <twice daily	10.9 (2.7, 19.1)	-6.6 (-12.7, -0.5)	-4.3 (-8.3, -0.4)	7.5 (0.9, 14.1)	-3.9 (-8.7, 1.0)	-3.6 (-6.8, -0.4)	5.4 (-1.1, 11.8)	-4.0 (-8.8, 0.8)	-1.4 (-4.5, 1.7)
**Health-related factors**									
Body mass index									
Normal	Ref	Ref	Ref	Ref	Ref	Ref	Ref	Ref	Ref
Overweight	16.2 (9.3, 23.1)	-7.5 (-12.8, -2.2)	-8.8 (-11.9, -5.6)	14.8 (7.6, 21.9)	-5.7 (-11.1, -0.3)	-9.1 (-12.4, -5.8)	19.1 (12.2, 25.9)	-8.0 (-13.2, -2.8)	-11.1 (-14.4, -7.8)
Obese	50.8 (37.1, 64.5)	-27.1 (-37.3, -17.0)	-23.6 (-30.2, -17.1)	46.9 (36.5, 57.2)	-24.0 (-31.7, -16.4)	-22.8 (-27.6, -18.1)	49.6 (40.1, 59.1)	-25.9 (-32.8, -18.9)	-23.7 (-28.2, -19.2)
MCS (per 10-point decrease) [b]	2.8 (-1.2, 6.7)	-0.9 (-3.8, 2.0)	-1.9 (-3.9, 0.2)	3.6 (-0.3, 7.4)	-1.3 (-4.2, 1.6)	-2.3 (-4.0, -0.5)	4.7 (0.5, 8.8)	-1.3 (-4.5, 2.0)	-3.4 (-5.3, -1.5)
PCS (per 10-point decrease) [b]	10.4 (4.7, 16.1)	-4.8 (-8.9, -0.6)	-5.7 (-8.7, -2.7)	8.1 (3.6, 12.7)	-4.3 (-7.6, -0.9)	-3.9 (-6.0, -1.7)	11.4 (7.2, 15.5)	-5.0 (-8.0, -2.0)	-6.3 (-8.4, -4.3)
Number of chronic conditions [c] (per new condition)	11.0 (3.9, 18.1)	-7.4 (-12.6, -2.3)	-3.6 (-6.9, -0.2)	11.0 (6.6, 15.5)	-7.5 (-10.8, -4.2)	-3.6 (-5.7, -1.5)	6.2 (3.0, 9.4)	-4.2 (-6.5, -1.8)	-2.0 (-3.5, -0.4)

Abbreviations: CI: confidence interval; MCS: Mental component summary score from the Short Form 36 Health Survey; PCS: SF 36 Physical component summary score from the Short Form 36 Health Survey.

[a] estimated from linear regression adjusted for all covariates and waking time accounting for missing data using multiple imputation.

[b] Lower MCS and PCS correspond to poorer mental and physical summary score, respectively.

[c] Chronic conditions include hypertension, diabetes, coronary heart disease, stroke, heart failure, arthritis, cancer, depression, dementia, Parkinson’s disease, and chronic obstructive pulmonary disease.

© 2022 Chen M et al. *JAMA Network Open.*

eTable 6. Association of Exposure Factors With Outcomes Among 1328 Women Accounting for Missing Data Using Multiple Imputation

	20-year follow-up			9-year follow-up			Cross-sectional analyses		
	Time difference (95% CI) in: [a]			Time difference (95% CI) in: [a]			Time difference (95% CI) in: [a]		
	**SB**	**LIPA**	**MVPA**	**SB**	**LIPA**	**MVPA**	SB	**LIPA**	**MVPA**
**Socio-demographic factors**									
Age (per 5 years)	18.6 (12.9, 24.4)	-7.5 (-11.8, -3.1)	-11.2 (-14.0, -8.4)	19.0 (13.1, 24.8)	-7.3 (-11.7, -2.9)	-11.6 (-14.5, -8.8)	16.7 (10.9, 22.6)	-6.1 (-10.5, -1.6)	-10.7 (-13.5, -7.8)
Non-white ethnicity	9.1 (-10.7, 28.9)	-5.6 (-19.7, 8.6)	-3.5 (-13.2, 6.1)	5.1 (-13.9, 24.1)	-0.6 (-14.1, 13.0)	-4.6 (-14.1, 5.0)	7.6 (-11.1, 26.2)	-3.5 (-16.8, 9.9)	-4.1 (-13.5, 5.2)
Lower occupational position	-8.1 (-21.6, 5.4)	8.2 (-2.1, 18.4)	-0.0 (-6.4, 6.3)	-15.6 (-30.1, -1.0)	10.8 (-0.1, 21.7)	4.8 (-2.1, 11.7)	-15.22 (-29.1, -1.3)	11.6 (1.1, 22.0)	3.7 (-3.2, 10.6)
Not married/ cohabitating	15.0 (2.8, 27.3)	-13.8 (-22.9, -4.6)	-1.3 (-7.0, 4.4)	11.0 (-0.7, 22.8)	-9.7 (-18.9, -0.5)	-1.3 (-6.7, 4.0)	9.0 (-2.6, 20.5)	-11.9 (-20.8, -2.9)	2.9 (-2.5, 8.4)
**Behavioral factors**									
Smoking status									
Never smoker	Ref	Ref	Ref	Ref	Ref	Ref	Ref	Ref	Ref
Past smoker	-5.5 (-18.5, 7.6)	0.5 (-9.1, 10.1)	5.0 (-1.4, 11.4)	-2.3 (-14.3, 9.7)	-0.2 (-9.2, 8.8)	2.5 (-3.3, 8.4)	-4.0 (-15.7, 7.8)	1.8 (-7.0, 10.6)	2.2 (-3.6, 7.9)
Current smoker	10.9 (-7.2, 29.0)	-5.6 (-18.9, 7.7)	-5.3 (-14.1, 3.5)	4.3 (-17.0, 25.7)	2.7 (-13.3, 18.7)	-7.0 (-17.5, 3.5)	13.5 (-15.2, 42.3)	-4.8 (-26.1, 16.5)	-8.7 (-22.8, 5.5)
Alcohol intake									
None	10.2 (-4.0, 24.4)	-7.2 (-17.5, 3.1)	-3.0 (-10.0, 4.0)	13.4 (-0.9, 27.7)	-10.2 (-21.2, 0.8)	-3.2 (-9.8, 3.4)	6.2 (-7.0, 19.4)	-4.1 (-13.8, 5.6)	-2.1 (-8.6, 4.3)
Moderate	Ref	Ref	Ref	Ref	Ref	Ref	Ref	Ref	Ref
High	0.7 (-16.2, 17.7)	-0.4 (-13.1, 12.3)	-0.4 (-8.4, 7.6)	-4.0 (-18.9, 10.9)	-3.8 (-14.8, 7.2)	7.8 (0.4, 15.2)	-1.6 (-19.0, 15.7)	-6.6 (-19.6, 6.4)	8.3 (0.0, 16.5)
Fruits & vegetables intake <twice daily	10.1 (-2.9, 23.2)	-5.5 (-15.5, 4.4)	-4.6 (-10.7, 1.5)	7.7 (-5.0, 20.4)	-7.1 (-16.4, 2.1)	-0.6 (-6.8, 5.6)	4.5 (-7.9, 16.8)	-4.7 (-14.1, 4.8)	0.2 (-5.7, 6.1)
**Health-related factors**									
Body mass index									
Normal	Ref	Ref	Ref	Ref	Ref	Ref	Ref	Ref	Ref
Overweight	33.0 (20.1, 46.0)	-18.5 (-28.3, -8.6)	-14.6 (-20.7, -8.4)	24.5 (11.2, 37.8)	-10.8 (-20.6, -1.0)	-13.7 (-20.0, -7.3)	17.2 (3.8, 30.7)	-8.6 (-18.6, 1.5)	-8.7 (-15.0, -2.3)
Obese	45.2 (27.7, 62.8)	-26.8 (-40.4, -13.1)	-18.5 (-26.9, -10.0)	46.5 (31.3, 61.6)	-25.7 (-37.2, -14.2)	-20.7 (-27.7, -13.8)	52.1 (37.4, 66.8)	-31.2 (-42.4, -20.0)	-20.9 (-27.9, -14.0)
MCS (per 10-point decrease) [b]	5.6 (-0.5, 11.6)	-3.4 (-8.1, 1.2)	-2.1 (-5.1, 0.9)	4.6 (-1.0, 10.1)	-3.1 (-7.3, 1.0)	-1.4 (-4.2, 1.4)	4.7 (-0.9, 10.2)	-2.6 (-6.9, 1.7)	-2.1 (-4.9, 0.8)
PCS (per 10-point decrease) [b]	5.0 (-2.6, 12.6)	-0.1 (-5.7, 5.5)	-4.9 (-8.5, -1.2)	5.3 (-0.7, 11.3)	-2.0 (-6.5, 2.6)	-3.3 (-6.3, -0.3)	9.0 (2.9, 15.2)	-4.2 (-8.7, 0.4)	-4.9 (-7.9, -1.9)
Number of chronic conditions [c] (per new condition)	14.5 (0.8, 28.2)	-9.4 (-19.7, 1.0)	-5.2 (-11.4, 1.0)	13.2 (5.9, 20.4)	-9.8 (-15.5, -4.1)	-3.4 (-6.7, 0.0)	9.7 (4.3, 15.0)	-6.1 (-10.3, -2.0)	-3.6 (-6.2, -0.9)

Abbreviations: CI: confidence interval; MCS: Mental component summary score from the Short Form 36 Health Survey; PCS: SF 36 Physical component summary score from the Short Form 36 Health Survey.

[a] estimated from linear regression adjusted for all covariates and waking time accounting for missing data using multiple imputation.

[b] Lower MCS and PCS correspond to poorer mental and physical summary score, respectively.

[c] Chronic conditions include hypertension, diabetes, coronary heart disease, stroke, heart failure, arthritis, cancer, depression, dementia, Parkinson's disease, and chronic obstructive pulmonary disease.

© 2022 Chen M et al. *JAMA Network Open.*
